# Effective treatment options for musculoskeletal pain in primary care: A systematic overview of current evidence

**DOI:** 10.1371/journal.pone.0178621

**Published:** 2017-06-22

**Authors:** Opeyemi O. Babatunde, Joanne L. Jordan, Danielle A. Van der Windt, Jonathan C. Hill, Nadine E. Foster, Joanne Protheroe

**Affiliations:** Arthritis Research UK Primary Care Centre, Research Institute for Primary Care & Health Sciences, Keele University, Keele, United Kingdom; University of Bern, SWITZERLAND

## Abstract

**Background & aims:**

Musculoskeletal pain, the most common cause of disability globally, is most frequently managed in primary care. People with musculoskeletal pain in different body regions share similar characteristics, prognosis, and may respond to similar treatments. This overview aims to summarise current best evidence on currently available treatment options for the five most common musculoskeletal pain presentations (back, neck, shoulder, knee and multi-site pain) in primary care.

**Methods:**

A systematic search was conducted. Initial searches identified clinical guidelines, clinical pathways and systematic reviews. Additional searches found recently published trials and those addressing gaps in the evidence base. Data on study populations, interventions, and outcomes of intervention on pain and function were extracted. Quality of systematic reviews was assessed using AMSTAR, and strength of evidence rated using a modified GRADE approach.

**Results:**

Moderate to strong evidence suggests that exercise therapy and psychosocial interventions are effective for relieving pain and improving function for musculoskeletal pain. NSAIDs and opioids reduce pain in the short-term, but the effect size is modest and the potential for adverse effects need careful consideration. Corticosteroid injections were found to be beneficial for short-term pain relief among patients with knee and shoulder pain. However, current evidence remains equivocal on optimal dose, intensity and frequency, or mode of application for most treatment options.

**Conclusion:**

This review presents a comprehensive summary and critical assessment of current evidence for the treatment of pain presentations in primary care. The evidence synthesis of interventions for common musculoskeletal pain presentations shows moderate-strong evidence for exercise therapy and psychosocial interventions, with short-term benefits only from pharmacological treatments. Future research into optimal dose and application of the most promising treatments is needed.

## Introduction

Pain as a result of musculoskeletal problems of the back, neck, shoulder, knee and multi-site pain is an increasing cause of diminished quality of life, and increased demands on healthcare [1–3]. Prognosis is often poor with many people reporting persistent symptoms 6 to 12 months after consulting their primary care practitioner [4, 5]. Furthermore, the likelihood of persistent or recurrent clinical symptoms may accentuate the physical, psychological, and socio-economic impacts of musculoskeletal pain.

Musculoskeletal pain is managed by a plethora of treatment options, most delivered in primary care by first contact clinicians such as general practitioners, physiotherapists, chiropractors and osteopaths. These include non-pharmacological treatments (e.g. self-management advice and education, exercise therapy, manual therapy and psychosocial interventions), complementary therapies (e.g. acupuncture), and pharmacological interventions (e.g. analgesics, non-steroidal anti-inflammatory drugs (NSAIDs), corticosteroid injections). For those with refractory symptoms, surgical interventions (e.g. arthroscopic debridement, total knee replacements, and laminectomies) may be considered. However, for the overarching aim of reducing pain and improving function, recommendations are equivocal in respect to the effectiveness of various treatment options that are used across a range of common musculoskeletal pain presentations. For example, evidence for the effectiveness of corticosteroid injections for relief of shoulder or knee pain is inconsistent [6, 7]. Similarly, the efficacy and safety of simple analgesics and NSAIDs for reducing symptoms associated with osteoarthritis and back pain is uncertain [8–11]. In order to provide optimal care to patients with musculoskeletal pain and ensure the efficient use of healthcare resources, a comprehensive overview of the available evidence for the most effective treatment options for musculoskeletal pain presentations is essential.

Evidence from trials and systematic reviews indicate that most treatments for musculoskeletal pain provide small to moderate short-term benefits, with a lack of evidence for long-term effectiveness [12]. Also, there appears to be a wide heterogeneity in the response of patient symptoms to treatments, suggesting that some patients may benefit more from some treatments than others [12]. Due to an apparent lack of information on the comparative effectiveness of available treatment options, there is a need to summarise current evidence regarding the best treatments for musculoskeletal pain presentations.

Previous reviews and guidelines that describe the effectiveness of treatments for musculoskeletal pain specifically focus on single regional pain sites, such as shoulder pain [13, 14], knee pain [15, 16] or low back pain [17–20]. However, research evidence suggests that in the general population and those presenting to primary care, localised musculoskeletal pain frequently coexists in more than one body region [21, 22] and that those with different regional pains share similar underlying attributes, course of symptoms and prognostic factors [23, 24]. Nevertheless, for many patients, clinical decision-making regarding treatment is often focussed on the specific body region without much recourse to the potential influence of prognostic factors or other co-existing pain problems. As a result, informed choices about which treatment might work best for which individual also remain a substantial clinical challenge. A more holistic view is perhaps difficult to obtain since trials and systematic reviews usually focus on a specific musculoskeletal pain site, comparing only two or three treatment options. To our knowledge there are no published reviews in which evidence regarding the comparative effectiveness of a wide range of treatments is systematically synthesised for the most common musculoskeletal pain presentations.

The aim of this study was to critically appraise current best evidence regarding the effectiveness of treatments to reduce pain and /or improve function for people with the five most common musculoskeletal pain presentations in primary care (i.e., back, neck, shoulder, knee and multi-site pain as indicated by Jordan et al.[25]). The specific objectives of this review were to:

1. identify effective treatment options for the five most common musculoskeletal pain presentations and
2. highlight gaps in evidence and priorities for policy or future research.

The review also identified, where available, evidence regarding patient subgroups most likely to respond to different treatment options.

## Methods

### Sources of data and search strategy

Integrated information from higher levels of evidence has been suggested as an “ideal source of evidence for clinical decision-making” by the Evidence Based Practice group (http://hsl.mcmaster.libguides.com/ebm). Therefore, using national clinical guidelines, policy documents, care pathways such as Map of Medicine (MoM), and clinical evidence summaries as a starting point, the search for evidence for this overview followed a pyramidal tract through a hierarchy of available evidence. Sources of evidence for the overview included: Clinical Knowledge Summaries, Map of Medicine, TRIP Database (systematic reviews and clinical guidelines), the Cochrane Library (including Cochrane database of systematic reviews, Database of abstracts of reviews of effects, Health technology assessment database), MEDLINE and EMBASE (using specific search filters to retrieve systematic reviews and clinical guidelines), reference lists of included systematic reviews and guidelines, research stakeholders and experts in the field of musculoskeletal research. Evidence sources were initially accessed in January 2014 and regularly checked for new updates at eight week intervals through to March 2015 whilst the review was ongoing. A Cochrane library search update was conducted in February and August 2016 in order to identify newly published Cochrane reviews.

All Cochrane reviews matching the inclusion criteria were included in the synthesis. Relevant non-Cochrane systematic reviews were added where there were no (up-to-date) Cochrane reviews summarising the effectiveness of a particular treatment. Additional searches of the bibliographic databases, MEDLINE and EMBASE (using narrow or specific search filters to retrieve systematic reviews and clinical guidelines) were carried out to identify and retrieve (1) relevant systematic reviews, and (2) more recently published relevant RCTs that had not yet been summarised in reviews or guidelines or where evidence gaps clearly existed. For the bibliographic database searches, retrieved search results were limited to published articles from 2000 until December 2014 initially, and then updated in August 2016. The search strategy and search terms for these additional searches are profiled in supplementary S1 File.

Relevant publications (guidelines, systematic reviews and meta-analyses of RCTs as well as recent RCTs which are yet to be summarised in reviews) were obtained and assessed against predefined eligibility criteria according to the study protocol by two reviewers.

#### Inclusion criteria

- Study populations: Reviews/studies of adults (18 years and over) presenting with at least one of the five most common musculoskeletal pain presentations: back, neck, shoulder, knee and multi-site pain (the latter defined as musculoskeletal pain in more than one area of the body).
- Type of treatments: Reviews/studies of currently available treatments (including self-management advice and education, exercise therapy, manual therapy, pharmacological interventions (oral and topical analgesics, local injections), aids and devices, and other treatments (ultrasound, TENS, laser, acupuncture, ice / hot packs)) for musculoskeletal pain patients consulting in primary care were considered. Referral options for psychosocial interventions (such as cognitive-behavioural therapy and pain-coping skills) and surgery were also included. Comparison groups could include usual care, no intervention or other active interventions.
- Outcomes: Reviews/studies had to report outcomes of pain (e.g. intensity, widespreadness, bothersomeness, number of episodes, duration), and/or functional disability. These were considered primary outcomes for this review. Secondary outcomes such as psychological well-being / depression, catastrophising, quality of life (QOL), work related outcomes (e.g. sickness absence, return to work, days off work), and cost of treatment were highlighted, but were not required for inclusion in the review.

#### Exclusion criteria

- Narrative reviews, letters, editorials, commentaries, and meeting abstracts were excluded, as were biomechanical, laboratory studies, animal studies as well as previous RCTs that were already summarised in included reviews, cohort, case-control, and cross-sectional studies.
- Reviews/studies published in other languages than English.
- Reviews/studies of musculoskeletal pain populations with suspected serious pathologies (e.g. suspected fracture, cancer, cauda equina syndrome), inflammatory arthritis, crystal disease, spondyloarthropathy, polymyalgia rheumatica, whiplash injuries, pregnancy-related pain problems, and vulnerable patients (e.g. experienced significant recent trauma, cognitive impairment, dementia, terminal illness).

### Quality appraisal

In order to weigh the conclusion of reviews within our evidence summaries, the methodological quality of non-Cochrane systematic reviews was assessed using the 11-item ‘assessment of multiple systematic reviews’ (AMSTAR) checklist [26]. The guidelines and care pathways which were included in this evidence synthesis were not quality assessed as they all made use of published development processes based on explicit methodology.

### Extraction of data

Data were extracted by one reviewer using a data collection form and independently checked for consistency and completeness by a second reviewer. Clarifications were sought where needed and disagreements between reviewers resolved by discussion. Data were extracted on the effectiveness of non-pharmacological, pharmacological and surgical treatments for each musculoskeletal pain presentation separately, and where available, guidance or conclusions regarding patient subgroups mostly likely to respond to specific treatments. More specifically, data were extracted regarding:

- population characteristics (e.g. age, gender, symptom duration, musculoskeletal pain site and where possible musculoskeletal pain condition/diagnoses,
- treatments (type/intensity/dosage),
- primary and secondary outcome measures (as stated above),
- estimates of treatment effect (where pooled, and as presented in the systematic reviews),
- estimates of treatment effect for patient subgroups (where available),
- treatment setting (e.g. primary care), and
- sources of evidence.

Treatments were assessed for short-term (up to 3 months) and long-term (greater than 6 months) effectiveness based on the primary outcomes of pain and function.

### Grading of evidence

Summaries of the overall evidence for the effectiveness of treatment options and strength of recommendations for each pain site were assessed based on (a modified) GRADE rating (http://www.gradeworkinggroup.org/). Summary evidence from all included reviews and guidelines were graded, taking into account the:

- Primary sources of data (e.g. guidelines, systematic reviews, RCTs): expert opinion or consensus in guidelines was rated as very weak evidence, while RCTs, systematic reviews and evidence-based guidelines were graded as higher level of evidence
- Quality of systematic reviews (Cochrane reviews or high methodological quality as assessed by AMSTAR checklist)
- Magnitude of effect where a standardised mean difference (SMD) of 0.2 was considered small, 0.5 as medium, and 0.8 as large according to Cohen [27], and for binary outcomes success rate, relative risk (RR) >2 was considered a medium to large effect size [28]
- Level of precision (confidence interval and level of significance; p<0.05)
- The consistency of results across systematic reviews or RCTs.

For each treatment option, evidence was graded as:

1. “Very weak evidence”—based solely on expert opinion or consensus in guidelines only or in the absence of systematic review evidence
2. “Limited evidence”—in the presence of little evidence from systematic reviews/evidence-based guidelines AND when there were small, inconsistent, or non-significant treatment effect sizes
3. “Moderate evidence”–in the presence of little evidence from systematic reviews/evidence-based guidelines (as in 2) but showing a medium to large treatment effect OR in the presence of strong evidence from high quality systematic reviews, but with small or inconsistent treatment effect sizes
4. “Strong evidence”—in the presence of strong evidence from high quality systematic reviews and evidence-based clinical guidelines AND medium or large effect sizes.

Each summary of evidence / analysis was graded using the adapted GRADE criteria as described above and a narrative synthesis was subsequently presented, indicating the strength of the evidence as very weak, limited, moderate, or strong.

### Evidence synthesis

A narrative synthesis approach was undertaken. Given expected heterogeneity of sources of evidence, treatment settings, and the wide remit of this review (which covered currently available treatments in primary care and referral options for one or more musculoskeletal pain presentation), the evidence was summarised at a high level (using systematic reviews and guidelines where available), and therefore no new meta-analyses were conducted. However, pooled estimates of treatment effectiveness from systematic reviews, as well as comments on the consistency and magnitude of treatment effects were extracted and reported. Additional information from policy documents and guidelines on treatment recommendations and priorities, including the type of evidence from which it was generated (i.e. whether from RCTs, systematic reviews or expert opinion) was also noted. The gathered evidence was included in summary tables (S1–S7 Tables) to enable (indirect) comparisons to be made across pain sites for the various treatments. Gaps in the evidence were noted where no guidelines, systematic reviews or RCTs were found.

## Results

### Search results

**Fig 1:**
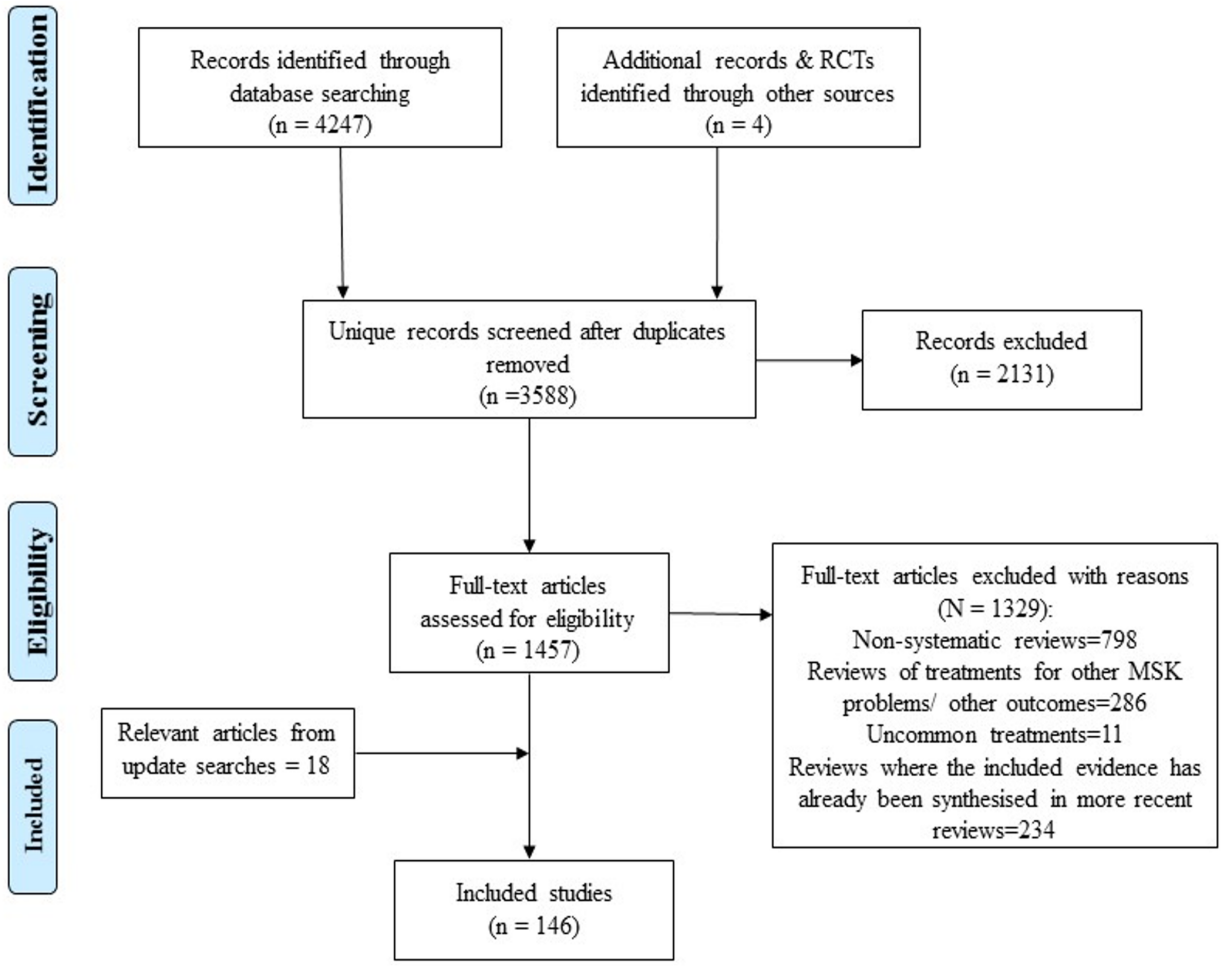
Review flow diagram (PRISMA).

A total of 3,588 unique citations (including Cochrane reviews) were retrieved from the electronic bibliographic databases. On assessing titles, abstracts and full texts against the inclusion criteria, 71 Cochrane systematic reviews met the selection criteria and were included. Non-Cochrane systematic reviews (n = 75) were only included where a gap not already covered by Cochrane reviews was identified, or if they represented new research that had not yet been considered within updated guidelines and care pathways. The remaining papers were excluded because they were not a systematic review (n = 798), focused on an area already covered by one of the included Cochrane systematic reviews (n = 234), were duplicate publications or did not fit the inclusion criteria (n = 2131). A summary of the review process outlining the selection of evidence is presented in Fig 1.

### Quality appraisal

**Fig 2:**
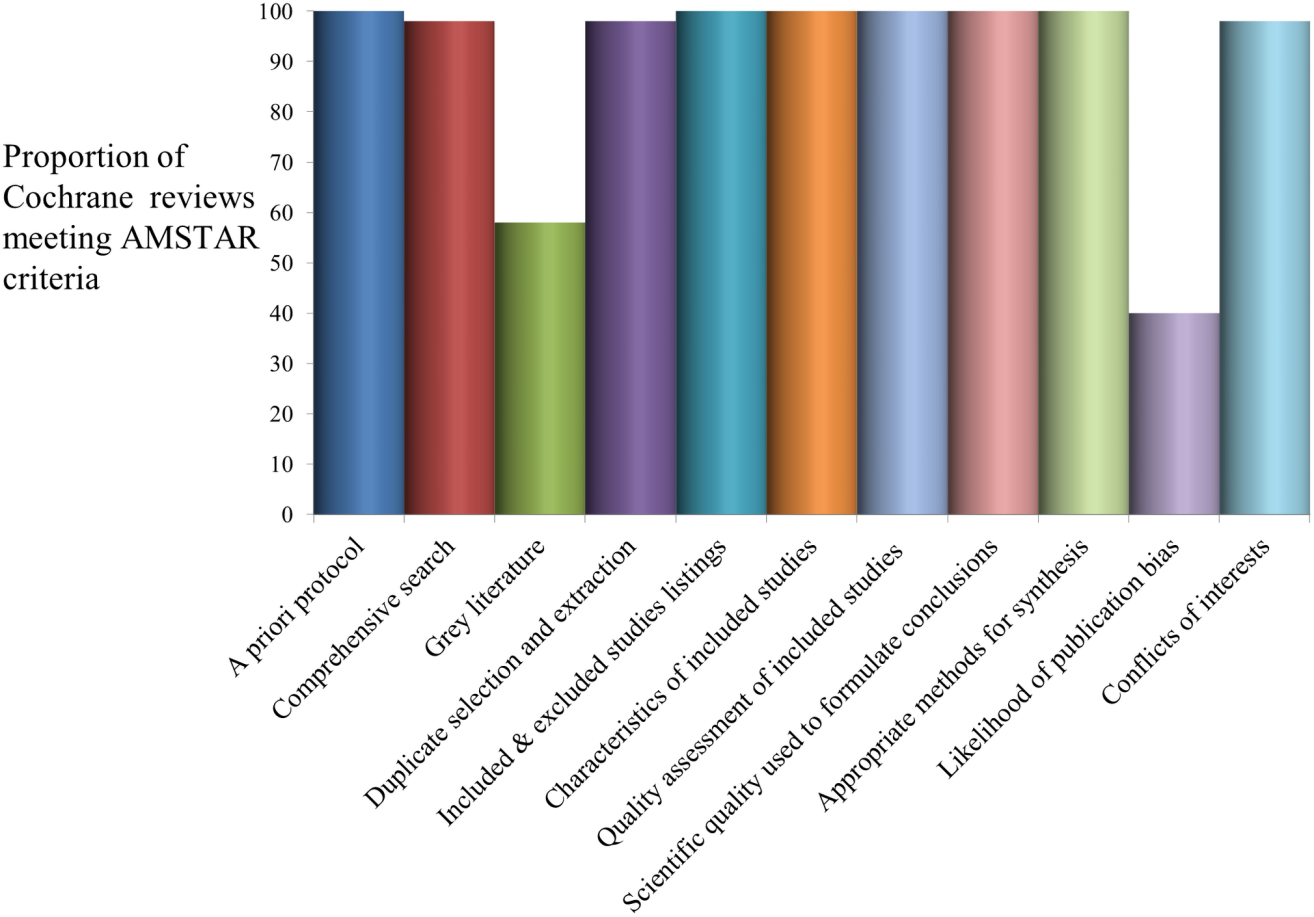
Quality assessment of contributing evidence from Cochrane reviews.

**Fig 3:**
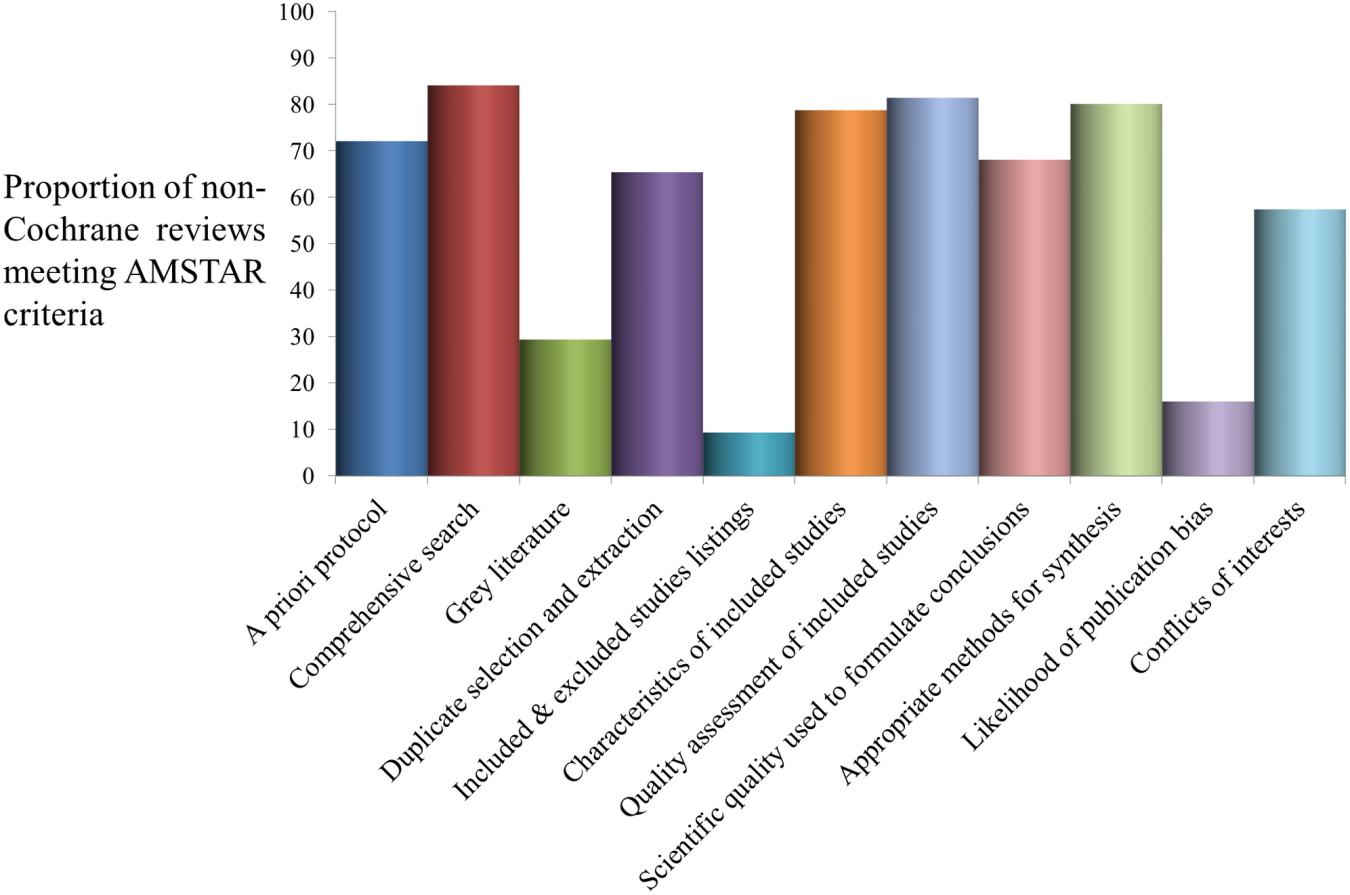
Quality assessment of contributing evidence from non-Cochrane systematic review & meta-analyses.

As Cochrane reviews followed a generic protocol specifying methods and review protocols go through a comprehensive peer review process prior to publication, the methodological quality of most Cochrane reviews included in this evidence synthesis was satisfactory (Fig 2). Cochrane reviews had flaws mainly associated with lack of searches for grey literature and/or no formal assessment of publication bias (Fig 2). As shown in Fig 3, methodological quality was less strong for non-Cochrane reviews, especially in terms of the comprehensiveness of the search strategy (including searches for grey literature), and listing of excluded studies (10%). Most reviews (81%) carried out some form of quality appraisal of included studies but study quality was not always incorporated into the evidence synthesis nor appropriately used to formulate conclusions (68%). Over half of the reviews (≈ 65%) minimised the risks of reviewer error and bias via duplicate processes for study selection and data extraction; and a very low proportion of reviews (16%) assessed the likelihood of publication bias.

### Included reviews and guidelines

Our searches identified reviews, guidelines and care pathways that covered a large range of treatment options for each musculoskeletal pain presentation. Based on specific review questions, authors of each review used particular criteria for identifying relevant trials in terms of setting, participants and interventions, resulting in variation across reviews in terms of the musculoskeletal condition, type and number of trials included, interventions, and reported outcomes of the reviews. A detailed description of the settings, populations, treatments and outcomes is provided in S1–S7 Tables. Each of the pain presentations (back, neck, shoulder, knee, multi-site pain) include several diagnostic categories, which are also summarised in the supplementary evidence tables.

### Evidence synthesis

Effectiveness of available treatments for musculoskeletal pain was highlighted in the following order: self-management advice and education, exercise therapy, manual therapy, pharmacological interventions, aids and devices, other treatments (including ultrasound, TENS, laser, acupuncture, ice / hot packs), psychosocial interventions and surgery. A summary of the findings is presented in Table 1. The overall grade of evidence from included reviews and guidelines on effectiveness of treatment options was fairly consistent for each of the five musculoskeletal pain presentations. For instance, the strength of evidence in support of the beneficial effects of exercise therapy for all the five musculoskeletal pain sites ranged between moderate and strong whilst there was generally limited evidence for low to medium effectiveness of manual therapy across the pain sites. There was wide variability in terms of the application and mode of delivery of even the same treatments. Within guidelines, there was little evidence regarding specific patient subgroups and predictors of response to treatments. However, any information extracted, regarding patient subgroups most likely to respond to specific treatment options is summarised in the evidence tables (S1–S7 Tables).

**Table 1:** Summary of findings.

*Evidence on treatment options across regional musculoskeletal pain presentations*						
Treatment Options	Evidence base	Regional pain	Outcomes *Pain* *Function Disability* *& other 2* ^*0*^ *Outcomes*	Magnitude of Effects	Strength of evidence (Grade)	
***Self-management advice & education***	2 clinical guidelines, 1clinical pathway, 8 reviews.	Back, neck, shoulder, knee & multi-site pain.	Pain Function	Small effect sizes (e.g. -3.2 points (95% CI -5.1, -1.3) on a 0–100 scale for back pain, Oliveira et al. 2012). Beneficial effects not proven in the long term.	**Limited evidence	
***Psychosocial interventions***	2 guidelines, 1 clinical pathway, 10 reviews & 2 RCTs.	Back, neck, shoulder, knee & multi-site pain. Limited amount of evidence on shoulder & knee pain.	Pain Function Quality of life	Medium to large effect sizes (e.g. MD -5.18; 95% CI -9.79 to -0.57, Henschke et al. 2011) for pain on a scale of 1 to10). Beneficial effects demonstrated in short & long term.	***Moderate evidence	
***Exercise Therapy***	4 guidelines, 3 policy documents, 32 reviews, 1 RCT.	Back, neck, shoulder, knee & multi-site pain.	Pain Function Quality of life Work-related outcomes.	Medium to large summary effects sizes (e.g. SMD 0.65, 95% CI: -0.09 to 1.39 for multi-site pain, Busch et al 2007, & RR 7.74, 95% CI: 1.97 to 30.32 for shoulder pain, Green et al 2003) Beneficial effects in the short & long-term for all five pain presentations.	****Strong evidence	
***Manual therapy***	3 guidelines & 21 reviews.	Back, neck, & shoulder pain.	Pain Function	Small effect sizes (e.g. NNT 5, for neck pain, Gross et al. 2012, & MD: -4.16, 95% CI -6.97 to -1.36, on 0–100 point scale for back pain, Rubinstein et al. 2011). Short-term effect on chronic pain but no strong evidence of long-term effectiveness compared to other standard treatments.	**Limited evidence	
***Pharmacological Treatments—(oral & topical analgesics)***	3 guidelines, 1 clinical pathway & 30 reviews.	Back, neck, shoulder, knee & multi-site pain.	Pain Function Evidence on function less often reported.	Medium effect sizes (e.g. NNT 4.6 (95% CI 3.8 to 5.9 for NSAIDs compared to placebo, Mason et al. 2004). Cox-2 selective inhibitors and opioids reduce pain in the short-term but the risk of adverse effects such as gastrointestinal bleeding and opioids-induced hyperalgesia needs careful consideration.	***Moderate evidence	
***Pharmacological Treatments–(Corticosteroid injections)***	3 guidelines, 1 clinical pathway & 16 reviews.	Back, neck, shoulder, & knee pain. Limited effects on back and neck pain.	Pain	Medium to large effect sizes (e.g. RR: 3.11 (95% CI 1.61 to 6.01 using injections for relieving moderate to severe knee pain in the short term compared to placebo, Belamy et al 2006).	****Strong evidence	
***Other treatments (Aids*, *Devices*, *complementary /alternative therapy)***	5 guidelines, 1 clinical pathway, 1 policy document, & 20 reviews.	Back, neck, shoulder & knee pain.	Pain Function	Small, non-significant or inconsistent, summary effect sizes.	**Limited evidence	
***Surgery***	1 guideline, clinical pathway document, 17 reviews.	Back, neck, shoulder, knee & multi-site pain.	Pain Function Quality of life	Effect sizes (not often estimated). Beneficial effects on pain & function in the short term with little empirical evidence for sustained long-term improvement.	**Limited evidence	

*Very weak evidence: Expert opinions or consensus in guidelines only / Absence of evidence in a single systematic review.

** Limited evidence: Little empirical evidence from systematic reviews/evidence-based guidelines AND when there were small, inconsistent, or non-significant treatment effect sizes.

*** Moderate evidence: little empirical evidence from systematic reviews/evidence-based guidelines (as in limited evidence) but showing a medium to large treatment effect OR in the presence of strong empirical evidence from high quality systematic reviews, but with small or inconsistent treatment effect sizes across systematic reviews.

**** Strong evidence: Strong empirical evidence from high quality systematic reviews and evidence based clinical guidelines AND medium or large effect sizes.

#### Self-management advice and education

Evidence base: Evidence was extracted from two clinical guidelines, one clinical pathway and eight reviews about the effectiveness of self-management advice and education. As assessed by AMSTAR, the methodological quality of systematic reviews was moderate or high but the primary studies within those reviews were generally low or moderate in quality. Given mostly in the form of oral and / or written information, advice and education was directed at improving patients' understanding of their musculoskeletal pain, and self-management techniques, addressing patients’ concerns about serious causes and outcomes, supporting return to function, and minimising dependence on healthcare providers [20, 29–38].

Magnitude of effects: Self-management advice and education was typically provided to either individuals or patient groups, as part of an intervention programme and were not tested in isolation against a control treatment (S1 Table). Therefore, the evidence for the effectiveness of self-management advice and education alone on the outcomes of pain and function was difficult to assess. Where estimated, summary effect sizes were usually small and/or not statistically or clinically significant. For instance, for back pain patients who received self-management advice, Oliveira et al [37] reported a pooled Mean Difference (MD) at short-term (up to 3 months) follow-up for pain of -3.2 points on a 0–100 scale (95% CI, -5.1 to -1.3) and of -2.3 points (95% CI, -3.7 to -1.0) for function. There was no evidence regarding patient subgroups most likely to respond to self-management advice and education.

Strength of evidence: The evidence for self-management advice and education supporting expert opinion in clinical guidelines and consensus meetings as well as systematic reviews showed small effects on pain and function. Pooled results from meta-analyses tended to have wide confidence intervals although recommendations for the use of advice and education were consistent. Overall strength of evidence was graded as limited.

Bottom line: Despite the limited evidence-base, there were strong recommendations for the use of self-management advice and education as a first line treatment option for musculoskeletal pain.

#### Exercise therapy

Evidence base: Synthesized evidence for the effectiveness of exercise on musculoskeletal pain included 10 Cochrane reviews [13, 18, 39–46], four guidelines [16, 20, 29, 47] and three policy documents [38, 48, 49]. Evidence from other reviews (n = 22), [36, 50–71] and one additional trial [72] were also considered. Quality of reviews ranged from moderate to high.

Magnitude of effects: Exercise therapy was determined to be beneficial for pain, function and quality of life in all five pain presentations [13, 16, 18, 20, 29, 36, 38, 47, 55, 56, 58–60, 63]. See supplementary S2 Table. Reviews and guidelines on exercise for neck pain [38, 58, 66] generally found exercises to be beneficial for function but no pooled estimates were provided. Exercise therapy led to clinically significant improvements in pain, function and quality of life for shoulder, knee, back and multi-site pain. In addition, medium to large summary effect sizes were reported in favour of exercise across the body of evidence, for example; RR 7.74; CI, 1.97 to 30.32 and RR 1.53; CI, 0.98 to 2.39 for improvement of shoulder pain and function respectively [13]; MD -1.46, CI -2.39 to -0.54 on a scale of 0 to 10) for pain as well as function (SMD 1.10, 95% CI, 0.58 to 1.63) for knee pain [46]; and MD 7.3 (95% CI, 3.7 to 10.9 points on a scale of 0–100) for low back pain [18]. With respect to multi-site pain[41], aerobic exercises was found to lead to improvement in global well-being (SMD 0.49, 95% CI, 0.23 to 0.75), physical function (SMD 0.66, 95% CI, 0.41 to 0.92) and pain (SMD 0.65, 95% CI, -0.09 to 1.39).

There appears to be little empirical evidence in favour of any particular exercise type, programme or mode of delivery, either as structured individual or group treatment for musculoskeletal pain [18, 39, 41, 48–50, 57, 65, 66, 68–70, 72, 73], although functional exercises (which adapt patients’ exercises to their activities of daily living, and enables them to perform such activities more easily and without injuries) appear to be more beneficial than exercises not specifically targeting function. There was no evidence regarding patient subgroups most likely to respond to exercise therapy. While some contributing reviews included information on whether patient symptoms were acute and chronic, it was difficult to assess if any particular exercise therapy had better effects on acute or chronic symptoms.

Strength of evidence: On the basis of medium to large summary effects sizes from high quality reviews, and clinical guidelines, the strength of evidence for the effectiveness of exercise therapy for pain, function, and quality of life for patients with musculoskeletal pain, was graded as strong.

Bottom line: Current evidence shows significant positive effects in favour of exercise on pain, function, quality of life and work related outcomes in the short and long-term for all the musculoskeletal pain presentations (compared to no exercise or other control) but the evidence regarding optimal content or delivery of exercise in each case is inconclusive.

#### Manual therapy

Evidence base: Six Cochrane reviews [44, 45, 74–77], three guidelines [20, 38, 78], and 15 other systematic reviews [54, 58, 66, 79–90] contributed to the evidence synthesis on the effect of manual therapy for the five most common musculoskeletal pain presentations. The effects of manual therapy on pain and function were mostly examined in combination with other treatments and mostly for non-acute pain. Methodological quality of reviews was moderate or high, although as highlighted in many of the reviews, a number of the primary trials on which reviews were based were of low quality.

Magnitude of effects: Pooled estimates for the effectiveness of manual therapy for musculoskeletal pain were generally statistically significant, but variable in terms of size of the treatment effect S3 Table. Manipulation, mobilisation and massage (where indicated) were reported to be beneficial for immediate and or short-term (4–6 weeks) improvement in range of motion and function in both acute and chronic neck pain patients as well as those with whiplash [38, 58, 66, 78, 91]. For instance, thoracic manipulation was found to lead to significant pain reduction (number needed to treat (NNT) 7), and increased function (NNT 5) in acute neck pain patients whilst a single session of thoracic manipulation was reported to result in immediate pain reduction for chronic neck pain patients (NNT 5) compared to placebo [44]. In a recent Cochrane review of manual therapy for adhesive capsulitis, 46% of participants reported treatment success with manual therapy and exercise compared with 77% who had corticosteroid injections (summary RR 0.6, 95% CI, 0.44 to 0.83), with an absolute risk difference of 31% (13% to 48%). The number reporting adverse events did not differ (summary RR 1.07, 95% CI, 0.76 to 1.49) between groups [45]. As with neck pain, manual therapy offers some benefits for range of motion and function in shoulder pain presentations [54, 79, 80, 82, 83, 89].

For back pain, evidence suggests that manual therapy alone or in combination with other treatments may offer some benefit for pain and function [20, 81, 85, 88]. Most authors presented no pooled estimates of treatment effects due to large heterogeneity among included trials. Where presented, summary effect sizes were generally small compared to no manual therapy or other control (e.g. SMD -0.25 (95% CI, -0.46 to -0.04 for pain and SMD -0.22, (95% CI, -0.36 to -0.07 for function) with negative SMD indicating lower levels of pain or functional limitation for manual therapy) in the short term [75, 76]; and (MD -0.46 (95% CI, -1.18 to 0.26 on a scale of 0 to 10) for pain in the long term [76]. Compared with other treatments (e.g. general practitioner care, acupuncture, ultrasound, standard physiotherapy, analgesic therapy, exercise, or back school), manual therapy appears to confer little or no clinically important effect on pain intensity, functional status, global improvement or return to work among patients with acute, subacute or chronic back pain with or without sciatica [74–77, 92, 93]. Type and experience of professional delivering the therapy did not show any clinically significant effect of on musculoskeletal pain [73]. There was low quality evidence that the efficacy of manual therapy might differ for subgroups of patients, with manual therapy tending to be more effective for acute non-specific low back pain patients with mobility deficit [90].

Strength of evidence: Despite several high quality reviews examining the effects of manual therapy on pain and function for neck, shoulder and back pain, current evidence generally shows small summary effect sizes or concludes no clinical effectiveness of manual therapy compared to sham or other active treatments. Overall strength of evidence was graded as limited.

Bottom line: Current evidence regarding manual therapy is beset by heterogeneity across clinical trials. Due to paucity of high quality evidence, it is uncertain if the efficacy of manual therapy might be different for different patient subgroups or influenced by the type and experience of professional delivering the therapy. On the whole, available evidence suggests that manual therapy may offer some beneficial effect on pain and function but it may not be superior to other non-pharmacological treatments (e.g. exercise) for patients with acute or chronic musculoskeletal pain.

#### Pharmacological treatments—Analgesics (oral & topical)

Evidence base: Thirty systematic reviews of pharmacological interventions for musculoskeletal pain examined the effectiveness of analgesics (opioids and non-opioids) in the short and long-term as well as in acute and chronic pain presentations. Comparisons were against placebo [94, 95], other pharmacological agents [48, 49, 96–100], corticosteroid injections [101], and no treatment [102, 103]. A few comparisons were made with other treatments such as laser and acupuncture [7]. Over 60% of the reviews on oral and topical analgesics were of high methodological quality while the rest were moderate. Reviews highlighted that the quality of included primary trials ranged from low to high quality.

Magnitude of effects: Compared to placebo, acetaminophen (paracetamol) was not more effective (SMD 0.13, 95% CI, 0.04 to 0.22) for relieving knee and back pain [94, 100, 103]. NSAIDs and opioid analgesics (especially for acute pain) were generally found to be effective but beneficial effects were evident mostly in the short-term [7, 14, 16, 29, 38, 94, 104, 105]. Cyclooxygenase (Cox)-2 selective inhibitors (e.g. celecoxib), were found to be effective for musculoskeletal pain relief. However, these were more likely to be associated with higher risks of adverse cardiovascular and gastrointestinal events (hazard ratio 2.18, 95% CI 1.82, 2.61), compared to non-selective NSAIDs [48, 49]. In the long-term and for more chronic pain presentations, stepwise analgesia according to the WHO analgesic ladder (mostly based on expert opinion) may be recommended [20, 29, 106–109]. Medium effect sizes were commonly reported S4 Table. For instance, topical NSAIDs were found to be more beneficial compared to placebo with summary RR of 1.9 (95% CI, 1.7 to 2.2) and a NNT of 4.6 (95% CI, 3.8 to 5.9) in the short-term [98, 99, 110, 111]. Furthermore, duloxetine, commonly used for multi-site pain may be carefully considered where there has been inadequate clinical response to initial pharmacologic treatments [48]. The effects of analgesics for improving function were less often reported in included reviews and guidelines.

Strength of evidence: With consistent medium summary effect sizes reported across moderate to high quality systematic reviews and clinical guidelines, there is moderate evidence that pharmacological therapies are beneficial for the short-term relief of musculoskeletal pain. Overall strength of evidence was graded as moderate.

Bottom line: NSAIDs, Cox-2 selective inhibitors and opioids reduce pain in the short-term, but the effect size is modest and the potential for adverse effects such as gastrointestinal bleeding and opioids-induced hyperalgesia need careful consideration.

#### Pharmacological interventions–injections

Evidence base: The evidence base for the effectiveness of injections for musculoskeletal pain involved the synthesis of three clinical guidelines [16, 112, 113] and one care pathway document [38], six Cochrane reviews [104, 114–118] and 13 other systematic reviews [7, 64, 66, 101, 119–127]. The systematic reviews were mostly high in methodological quality.

Magnitude of effects: A care pathway document [38], one guideline [49] and seven systematic reviews [64, 101, 116, 117, 119, 120, 125] supported evidence for the short-term (<4 weeks) benefits of corticosteroid injections for relieving moderate to severe shoulder pain (summary RR 1.43 (95% CI, 0.95 to 2.16) for corticosteroid injection compared with NSAIDs [119]). Likewise for knee pain, corticosteroid injections were found to be effective in the short-term for relieving moderate to severe pain compared to placebo ((summary RR: 3.11 (95% CI, 1.61 to 6.01); NNT 3 to 4) [115, 128]. Though corticosteroid injections were found to relieve pain, there was a lack of evidence for clinically significant effects on function [115]. For knee pain, viscosupplements such as intra-articular hyaluronate injections were found to be better than placebo (SMD 0.60, 95% CI 0.37 to 0.83) for reducing pain and improving function (SMD 0.61, 95% CI 0.35 to 0.87) in the short term (1–4 weeks). However, high clinical and statistical heterogeneity, evidence of publication bias and low quality trials preclude definitive recommendations about routine use in clinical practice [49].

Furthermore, the available evidence did not suggest injections are effective for the management of neck pain [66, 113, 121–124] or back pain [38, 104, 118]. Overall, there was no strong evidence for the use of epidural spinal injections with or without steroids, as benefits (immediate reductions in pain) were small and not sustained [114, 126, 127]. It appears the short-term pain relief offered by epidural spinal injections are hampered by significant heterogeneity, and that the severity and subtype of pathology may affect outcome [114, 126, 127].

Generally, in the long-term, injections may be no more effective than non-pharmacological interventions such as exercise [7, 64, 113, 116, 117, 119, 120, 125]. Evidence also suggests that the addition of corticosteroid injections to local anaesthetic does not confer improved symptom relief in the long-term [121, 122] however, expert opinion and guideline recommendations support its use prior to, or alongside, exercise and self-management advice [38, 64, 101, 112, 113, 119]. Although injections were often offered for acute pain relief and to enable patients to tolerate exercise therapy, there was no evidence regarding patient subgroups most likely to respond to injections.

Strength of evidence: Supported by high quality reviews, and clinical guidelines, medium to strong effect sizes across the various sources of evidence, injections offer clinically significant benefits for relieving moderate to severe shoulder and knee pain but in the short-term (up to 3 months) only. Overall, the strength of evidence was graded as strong.

Bottom line: The evidence indicates that injections offer short-term pain relief for shoulder and knee pain but effectiveness for back and neck pain is uncertain. Across the musculoskeletal pain presentations for which pharmacological injections may be given for pain relief, current evidence is equivocal on the optimal procedure (e.g. guided vs. unguided), frequency, dose and active component of the injections (though corticosteroid injections are more often reported in literature).

#### Aids & devices—Orthotics, tapes, braces, cervical collars and other support devices

Evidence base: The evidence for the effectiveness of aids and devices for pain and function included five guidelines [16, 20, 47, 112, 129], one clinical pathway [38], four Cochrane reviews [66, 77, 130–132], two best evidence syntheses [58, 133] and a meta-analysis [134]. The quality of reviews was moderate.

Magnitude of effects: Either as stand-alone treatment or mostly in combination with other treatments, aids and devices for musculoskeletal pain have generally shown small effects (see supplementary S5 Table) on pain, function or work outcomes [16, 20, 38, 58, 66, 77, 131–133]. Routine use of collars has not been found to confer any clinically significant benefits for neck pain [38, 58, 66, 133]. This may be attributed to marginal pain relief (in the short-term), and inclination to induce rest and inactivity hence prolonging disability. Patellar taping has been shown to have some beneficial effects (in the short-term) on pain and function in patients with patellofemoral pain [16, 20, 47, 112, 129]. Warden et al.[134] reported significantly less pain on a 100-mm scale (weighted mean difference (WMD) = -20.1, 95% CI, -26.0 to -14.3, p < .001) for patellofemoral pain patients treated with medially directed tape compared to patients treated with no tape or patients treated with sham tape (WMD = -13.3, 95% CI, -18.1 to -8.4, p < .001). There is very weak empirical evidence for the beneficial effects of knee braces but in grade II and III collateral ligament injuries, short-term (4–6 weeks) application of a hinged brace may be considered as part of rehabilitation [38, 47]. Empirical evidence suggests lumbar supports are not effective for improving pain and function in back pain patients [20]. This review did not find any evidence regarding specific patient subgroups for which aids and devices might be most beneficial.

Strength of evidence: Supported mostly by expert opinion or consensus in guidelines as well as small, inconsistent, or non-significant treatment summary effect sizes from systematic reviews, overall evidence for the use of aids and devices in the management of musculoskeletal pain is graded as limited.

Bottom line: For neck, shoulder, back and knee pain presentations, available evidence does not justify routine use of aids and devices for effective improvement of pain, function, and / or work outcomes.

#### Other treatments: Acupuncture, ultrasound, TENS, laser, ice / hot packs

Evidence base: Contributing evidence on the effectiveness of acupuncture, therapeutic ultrasound, TENS, laser, and superficial ice / hot packs for pain and function included five guidelines [16, 20, 47, 112, 129], one policy and one clinical pathway document [38, 48], 14 Cochrane reviews [13, 48, 76, 135–148] and 18 systematic reviews [36, 55, 59, 149–159]. The quality of reviews was mostly moderate with some reviews having high methodological quality. However, within the reviews and clinical documents, there was large heterogeneity and significant publication bias in primary studies.

Magnitude of effects: Compared to treatments such as analgesia, and exercise, these interventions have been less frequently evaluated, and the quality of RCTs is generally low. Also, for many of these treatments (i.e., therapeutic ultrasound, laser, and superficial ice / hot packs), reports of high clinical and methodological heterogeneity within the trials contributing to reviews preclude statistical pooling of effect estimates. There was also no evidence regarding specific patient subgroups which might benefit most from these treatments.

For acupuncture, available evidence from a good quality individual patient data meta-analysis suggests that acupuncture may be effective for short-term relief of back pain and knee pain with medium summary effect sizes (SMD 0.55 (95% CI, 0.51 to 0.58) and (SMD 0.42 (95% CI, 0.37 to 0.46)) respectively compared with usual care or no acupuncture [158]. However, effects on function were reported to be minimal and not maintained at longer-term follow-up [20, 139, 149, 152, 158]. Similarly for neck and shoulder pain, acupuncture was only found to be effective for short-term (immediately post-treatment and at short-term follow-up) symptom relief (SMD -0.37 (95% CI, -0.61 to -0.12)) and (WMD 3.53 (95% CI, 0.74 to 6.32 on a scale of 1–100)) compared to placebo [140, 148].

TENS was no more effective for reducing pain than placebo in chronic back pain [136, 141, 160, 161], neck pain [142], shoulder pain [145], knee [147] and chronic musculoskeletal pain [144, 150]. Ultrasound and shockwave therapy do not appear to significantly improve clinical outcomes for acute and chronic low back pain [162]. Also, for those with shoulder and/or neck pain, evidence suggests ultrasound does not confer significant or added benefit over placebo or other treatments [47, 55, 101, 140, 153, 157]. The evidence on effectiveness of laser therapy for shoulder pain [59, 159], or acute or chronic neck pain was inconclusive [151]. With regards to knee pain, other treatments including ultrasound, electromagnetic fields, low level laser therapy, TENS, biofeedback, neuromuscular electrical stimulation may confer added benefits to exercise and / or surgical treatment but empirical and clinical effect sizes are small and only supported by weak evidence [16, 47, 48, 112, 129, 143, 146, 147]. (Please refer to supplementary S5 Table for more details regarding other treatments).

Strength of evidence: There was little empirical evidence for the effectiveness of other treatments including ultrasound, TENS, laser, and superficial ice / hot packs. Presented summary effect sizes and estimates were often small, inconsistent, and non-significant. Although medium short-term effects were found for the effects of acupuncture on back and knee pain, overall strength of evidence was graded as limited.

Bottom line: The evidence for the clinical effectiveness of most of these other treatment options was not substantiated by strong evidence. Either as stand alone or in combination with other treatments, the often small effect sizes as a result of these treatments for improving musculoskeletal pain and function was mostly not clinically significant.

#### Psychosocial interventions

Evidence base: Evidence base for the effectiveness of psychosocial interventions (referred to various interventions such as cognitive-behavioural therapy and pain-coping skills, used to support people for overcoming challenges and maintenance of good health) included one guideline [20] and an overview of guidelines [163], one care pathway [38], four Cochrane reviews [164–168] and seven systematic reviews [17, 133, 169–173]. The quality of reviews ranged from moderate to high. Due to gaps in available systematic reviews of shoulder pain regarding psychosocial interventions, additional evidence from RCTs [174, 175] was extracted for shoulder pain.

Magnitude of effects: Reviews of psychosocial treatments for the management of musculoskeletal pain included a wide range of approaches that aimed to achieve increased self-management, behavioural and/or cognitive changes alongside biomedical management of pain S6 Table. Interventions were often multimodal and involved multidisciplinary treatment. At long-term follow-up, medium summary effect sizes (e.g. SMD 0.23; (95% CI, 0.43 to 0.040) compared to usual care and SMD 0.48 (95% CI, 0.93 to 0.04) compared to other active treatments [172]) were reported for pain, function and/ or other psychosocial related-outcome measures such as quality of life. With the exception of a few studies in back pain and neck pain, where patient recruitment and outcome reporting were based on targeted groups of patients receiving a psychosocial intervention according to baseline complexity of patients’ pain presentations [38, 164, 173], there was wide variability in the characteristics of patients included in trials. The effectiveness of psychosocial interventions for the management of shoulder, knee, and neck pain presentations was less well researched compared to those of back pain. Psychosocial interventions in combination with other treatment options appear to provide additional benefit for all musculoskeletal pain presentations. However, there was no consensus on specific treatment components, providers and settings for optimal outcomes [20, 38, 163–167, 170, 172–175]. Furthermore, methodological issues regarding primary studies reported by the systematic reviews, such as high attrition rates, incomplete outcome reporting, mixed treatment regimens and generally low sample numbers and patient heterogeneity made conclusions tentative.

Strength of evidence: Except for shoulder and knee pain, where the strength of evidence was limited, current evidence for the beneficial effects of psychosocial interventions for neck, back and multi-site pain is supported by moderate to high quality reviews, medium effect sizes with precise confidence intervals and this is consistent across sources of evidence. Overall, the strength of evidence was graded as moderate.

Bottom line: Available evidence suggests beneficial effects of psychosocial interventions, particularly for patients identified as having a poor prognosis prior to treatment. Also, outcome of psychosocial treatment appears to be influenced by other factors such as patient prognosis, the healthcare professional providing treatment, the settings for treatment delivery and the components of treatment.

#### Surgery

Evidence base: Evidence for the effectiveness of surgery for the musculoskeletal pain presentations (excluding multi-site pain) was synthesised from one guideline and a care pathway document [38, 47], nine Cochrane reviews [176–184] and eight systematic reviews [66, 185–191]. Reviews were mostly high in methodological quality.

Magnitude of effects: Most guidelines specify that surgical treatments are indicated in a small proportion of patients (as low as 8%) for neck, shoulder, back and knee pain presentations [38, 47, 181]. Within the body of synthesised evidence (supplementary S1 and S7 Tables), the presence of serious pathology, substantial pain and disability or symptoms which are refractory to conservative treatment were prominent indications for surgery [38, 47, 180, 191], but the roles of such factors in determining the long-term clinical outcome of treatment was equivocal [38, 180]. Based on clinical judgement and expert opinion, current evidence suggests early surgical intervention may be considered on a case by case basis [38, 47]. Generally for neck, shoulder, knee and back pain, when indicated, there is moderate evidence that surgical intervention does provide benefits for pain, and function compared to waiting list controls or conservative treatments including analgesia and exercise in the short-term [38, 66, 176, 178–180, 187]. In specific cases, such as arthroscopic debridement and joint lavage of the knee, available evidence indicates no clinically important benefit (SMD -0.11, 95% CI, to 0.42 to 0.21) for pain or function compared to control (SMD -0.10, 95% CI, -0.30 to 0.11) at three months [182]. Available evidence suggests there are no long-term benefits of surgical procedures for clinical outcomes compared with conservative treatment [177, 184–188, 190]. Neither was there strong evidence for a significant difference in favour of any particular surgical technique for any of the pain sites [182, 183, 189, 191].

Strength of evidence: Though reviews were mostly high in methodological quality, summary effect sizes were small. Overall strength of evidence of long-term effectiveness of surgery is limited except where directly indicated by specific serious pathology such as end-stage degenerative knee joint disease, persistent pain and functional limitation which are refractory to conservative treatments.

Bottom line: The effectiveness of surgery as a first line treatment option is not established in current literature. The current evidence base is limited in terms of quantity, especially comparing surgical versus conservative interventions but there is moderate evidence from guidelines, Cochrane reviews and other systematic reviews to support short-term efficacy of surgical interventions for pain and function for specific neck, shoulder, knee and back pain presentations. Available evidence also suggests that surgery is not superior to conservative treatment options in the long-term.

## Discussion

This review has systematically identified, synthesised and graded a large body of evidence on the effectiveness of treatment for musculoskeletal pain presentations. For most pain presentations, non-pharmacological treatments especially exercise therapy as well as psychosocial interventions, produced medium to large effects on pain and function, with corticosteroid injections potentially offering short-term benefit in those with knee and shoulder pain. NSAIDs and opioids (where appropriate) also offer short-term benefit for musculoskeletal pain, but the potential for adverse effects need careful consideration.

The effectiveness of exercise therapy, psychosocial interventions and corticosteroid injections was consistently supported by empirical evidence of mostly medium effect sizes provided by meta-analyses of RCTs, by guidelines, and expert opinion for musculoskeletal pain. With regard to intensity, and modes of applications of most treatments, the amount of clinical contact, the type of provider, setting, and delivery modes/techniques for effective treatment varied widely and, as yet, there is limited evidence to support choices regarding optimal delivery of these treatments. Therefore, further research to investigate the optimal dose and application of these treatment options is needed.

In this review, there was little information within the evidence base in relation to patient subgroups most likely to respond to different treatment options. Where available, for each treatment option, evidence regarding patient characteristics such as baseline pain severity and function, duration of pain, and previous pain episodes have been documented S1–S7 Tables. For most treatment options apart from manual therapy (due to low quality evidence for differential effects of manual therapy across patient subgroups), and psychosocial interventions (where moderate evidence supports targeting patient subgroups to psychosocial intervention according to baseline complexity), it is not certain if clinical outcomes for most treatment options may be improved by targeting patient subgroups. Given that there are many factors (including patient characteristics and risk of poor outcome) which may influence outcome of treatment, it is likely that, an optimal approach to management of musculoskeletal pain may involve strategic selection of treatments best suited for different patients. Future trials should be designed to bridge this gap in evidence for the management of musculoskeletal pain.

It is worth noting that in many of the reviews, guidelines and trials contributing to this evidence-base, individual treatments were rarely used in isolation. Therefore, the evidence for the isolated effectiveness of treatments in some reviews was difficult to assess. For instance, self-management advice and education was typically provided as part of intervention package rather than tested in isolation against a control treatment. Consequently, there was little empirical evidence about its effectiveness despite consistent support of the beneficial effects (by expert opinion and consensus in guidelines). This could impact the quality and level of evidence for the beneficial effects of otherwise promising treatments.

### Overall completeness and applicability of evidence

As expected, given the breadth of this review there was wide heterogeneity in study populations, outcomes, and statistical methods for estimating summary effect measures in the included systematic reviews. Interpreting findings within this overview was also complicated by variability in both the intervention and control conditions (placebo, no treatment, active treatments) examined within the reviews, making it difficult to summarise evidence regarding the magnitude of treatment effects. Furthermore, in this overview, the treatments provided in individual studies could not be described in detail; settings, exact content, intensity or dose of interventions may have varied; many interventions may have required specialist staff (e.g. injection, acupuncture, manual therapy, surgery) and the training and skills of providers are likely to have varied over time and locations. Control conditions were frequently not described in reviews and trials, and the definition of terms “routine care”, “standard care” or “no intervention” may vary depending on setting and country. In the conduct of this study however, concerted efforts were made to capture and report available contextual information when summarizing evidence regarding treatment effectiveness.

### Strengths and limitations of the review

Where possible, given the wide remit of this review, a number of steps were taken to ensure methodological rigour. The focus was on publications providing high quality evidence or recommendations, including Cochrane and other high quality reviews, well-developed clinical guidelines that met specific quality assurance criteria, and evidence-based multidisciplinary care plans as outlined in care pathways. For Cochrane reviews, all reviews used protocols that aimed to minimise bias whilst for non-Cochrane reviews, evidence of using systematic methods was a pre-requisite for inclusion in this study. In addition, separate structured and systematic searches of bibliographic databases were conducted to identify additional trials not covered in previous reviews, where gaps concerning the effectiveness of specific treatment options were identified.

This review provides evidence summaries regarding the effectiveness of a wide range of treatments for the five most common musculoskeletal pain presentations in primary care, drawing together findings from a large evidence base. To facilitate this rapid evidence summary, the methodology evolved as a rapid application of systematic review methods to synthesising evidence. Efforts were made to capture, appraise and synthesise the best available evidence in a systematic yet rapid fashion. Definitive elements of typical systematic review methods such as a comprehensive and systematic search of best available evidence, pre-specified inclusion and exclusion criteria, quality appraisal and synthesis have all been preserved. Further strengths of this review included independent assessment of eligibility for inclusion and data extraction for contributing reviews, data checks, appraisal of the quality of systematic reviews, and a standardised approach to synthesising evidence.

There are several limitations to this review. First, there was no independent assessment of the methodological quality of primary trials that were included in the reviews. As this is an overview of current best available evidence, methodological quality assessment of included primary studies depended largely on the ratings of systematic review authors rather than our own assessment of the details presented in the individual studies. The overview of evidence incorporating reviews of multiple interventions across many musculoskeletal pain conditions therefore may not follow strictly the process generally applied in a single systematic review of one intervention on a single target population. However, much care has been taken to ensure that our approaches to searching for evidence, quality appraisal and grading of available evidence, and synthesis (as highlighted in the methods section) were as rigorous and as transparent as possible.

In this overview, evidence on effectiveness of treatment options for musculoskeletal pain has been presented based on pain presentations at different body regions rather than on specific clinical diagnoses given available evidence of similarity of patient characteristics, prognosis and clinical course of musculoskeletal pain presentations irrespective of specific clinical diagnoses [23, 24]. However, information on the specific clinical diagnosis for which evidence was derived is indicated in the supplementary evidence S1–S7 Tables.

### Practice implications

Across health systems globally, there is wide variation in clinical management of musculoskeletal pain patients whereby the most effective treatment options are not consistently used, leading to inefficient care, unnecessary costs and in some cases harm [3, 192]. In a clinical field with so many treatment options, this summary of evidence provides patients, clinicians, managers, policy-makers, and researchers with a helpful “one stop” overview of the evidence for treatments.

In this review, despite an extensive search for evidence, there was a paucity of evidence on treatment for those with multi-site pain. This musculoskeletal pain presentation, often managed as chronic widespread pain and / or fibromyalgia has been less examined in the literature because effectiveness of most treatment options has traditionally been compared on a pairwise basis and according to individual regional pain presentations. However, regional pains are known to co-exist in individual patients [84]. Patients included in most of the studies addressing management of single site pain are likely to have pain in other sites as well. Hence future research needs to investigate interventions that address these multiple sites of pain, in order to better inform clinical practice.

The lack of information regarding patient subgroups most likely to respond to specific treatment options, equivocal recommendations on the optimal mode of treatments, as well as the obvious focus of treatment approaches on single pain sites rather than the individual with multi-site musculoskeletal pain are key specific gaps in the current body of knowledge identified in this review.

## Conclusions

Effective healthcare depends on high quality evidence. Best available evidence shows that patients with musculoskeletal pain problems in primary care can be managed effectively with non-pharmacological treatments such as self-management advice, exercise therapy, and psychosocial interventions. Pharmacological interventions such as corticosteroid injections (for knee and shoulder pain) were shown to be effective treatment options for the short-term relief of musculoskeletal pain and may be used in addition to non-pharmacological treatments. NSAIDs and opioids also offer short-term benefit for musculoskeletal pain, but the potential for adverse effects must be considered. Furthermore, the optimal treatment intensity, methods of application, amount of clinical contact, and type of provider or setting, are unclear for most treatment options.

## Supporting information

S1 File MSK systematic review search strategy. (DOCX)

S2 File PRISMA checklist. (DOC)

S1 Table MSK Self management advice & education. (DOCX)

S2 Table MSK exercise therapy. (DOCX)

S3 Table MSK manual therapy. (DOCX)

S4 Table MSK pharmacological therapy. (DOCX)

S5 Table MSK_other therapies. (DOCX)

S6 Table MSK psychosocial Rx. (DOCX)

S7 Table MSK surgery. (DOCX)
