# Supplementary material for: Effective treatment options for musculoskeletal pain in primary care: A systematic overview of current evidence
Source: PLoS One. 2017 Jun 22;12(6):e0178621. doi: 10.1371/journal.pone.0178621 (PMC5480856; doi:10.1371/journal.pone.0178621)
Supplement: S1 File — (DOCX) [file pone.0178621.s001.docx]

***STarTMSK_MEDLINE search strategy***

1. exp Back Pain/

2. ((back or spine or spinal) adj2 pain).ti,ab.

3. (lumbar$ or lumbo$).ti,ab.

4. Pain/ or pain.ti,ab.

5. 3 and 4

6. sciatica.ti,ab.

7. exp Sciatica/

8. 1 or 2 or 5 or 6 or 7

9. exp Shoulder Joint/

10. shoulder.ti,ab.

11. "rotator cuff".ti,ab.

12. glenohumeral.ti,ab.

13. subacromia$.ti,ab.

14. or/9-13

15. Pain/ or pain.ti,ab.

16. 14 and 15

17. exp Shoulder Pain/

18. "adhesive capsulitis".ti,ab.

19. Shoulder Impingement Syndrome/

20. or/16-19

21. ((neck or cervical or cervico$) adj2 pain).ti,ab.

22. exp Neck Pain/

23. Whiplash Injuries/

24. whiplash$.ti,ab.

25. or/21-24

26. Osteoarthritis, Knee/

27. (knee adj2 osteoarthritis).ti,ab.

28. Patellofemoral Pain Syndrome/

29. (knee or patell$).ti,ab.

30. Pain/ or pain.ti,ab.

31. 29 and 30

32. 26 or 27 or 28 or 31

33. Chronic Pain/

34. "joint pain".ti,ab.

35. Arthralgia/

36. "musculoskeletal pain".ti,ab.

37. "widespread pain".ti,ab.

38. "non-cancer pain".ti,ab.

39. "chronic pain".ti,ab.

40. or/33-39

41. Meta-Analysis as Topic/

42. meta analy$.tw.

43. metaanaly$.tw.

44. Meta-Analysis/

45. (systematic adj (review$1 or overview$1)).tw.

46. exp Review Literature as Topic/

47. 41 or 42 or 43 or 44 or 45 or 46

48. cochrane.ab.

49. embase.ab.

50. (psychlit or psyclit).ab.

51. (psychinfo or psycinfo).ab.

52. (cinahl or cinhal).ab.

53. science citation index.ab.

54. bids.ab.

55. cancerlit.ab.

56. "web of science".ab.

57. 48 or 49 or 50 or 51 or 52 or 53 or 54 or 55 or 56

58. reference list$.ab.

59. bibliograph$.ab.

60. hand-search$.ab.

61. relevant journals.ab.

62. manual search$.ab.

63. 58 or 59 or 60 or 61 or 62

64. selection criteria.ab.

65. data extraction.ab.

66. 64 or 65

67. Review/

68. 66 and 67

69. Comment/

70. Letter/

71. Editorial/

72. animal/

73. human/

74. 72 not (72 and 73)

75. 69 or 70 or 71 or 74

76. 47 or 57 or 63 or 68

77. 76 not 75

78. 8 and 77

79. 20 and 77

80. 25 and 77

81. 32 and 77

82. 40 and 77

83. exp practice guideline/

84. Health Planning Guidelines/

85. guideline*.ti.

86. (practice adj3 parameter*).ti,ab.

87. clinical protocols/

88. guidance.ti,ab.

89. care pathway*.ti,ab.

90. critical pathway/

91. (clinical adj3 pathway*).ti,ab.

92. algorithms/

93. consensus development conference.pt.

94. ("National Institute for Health and Care Excellence" or NICE).ti,ab.

95. (EULAR or "European League against Rheumatism").ti,ab.

96. (OARSI or "Osteoarthritis Research Society International").ti,ab.

97. (RCGP or "Royal College of General Practitioners").ti,ab.

98. (CSP or "Chartered Society of Physiotherapy").ti,ab.

99. or/83-98

100. 8 and 99

101. 20 and 99

102. 25 and 99

103. 32 and 99

104. 40 and 99

105. 78 or 100

106. 79 or 101

107. 80 or 102

108. 81 or 103

109. 82 or 104
