# Supplementary material for: Effective treatment options for musculoskeletal pain in primary care: A systematic overview of current evidence
Source: PLoS One. 2017 Jun 22;12(6):e0178621. doi: 10.1371/journal.pone.0178621 (PMC5480856; doi:10.1371/journal.pone.0178621)
Supplement: S1 Table — (DOCX) [file pone.0178621.s003.docx]

|  | | **Compendium of evidence on analysis of effectiveness of self-management advice and education and across regional musculoskeletal pain presentations** | | | | | | | |
| --- | --- | --- | --- | --- | --- | --- | --- | --- | --- |
| **Regional pain**  *(Sub-diagnosis)* | **Comparison (s)** | | **Specific patient profiles/ mediating risk factors**  *(e.g., pain severity @baseline; pain duration; previous pain episodes; age; movement restriction; baseline disability)* | **Outcomes**  *Pain*  *Functional Disability*  *& other 2 ^0^ Outcomes* | **Long term / short term** | **Results /Effect size** | **Specific Diagnostic considerations** | **Grade of evidence** | **Comments / summary of evidence** |
| **Neck Pain**   - *Whiplash injury/ Whiplash associated disorders (WAD)* - *Non-specific neck pain* - *Acute torticollis* - *Cervical radiculo-pathy.* | No intervention/ Usual care | | Acute pain conditions | Reduction in pain and disability Improvement in function | Mostly in the short term. Current evidence base has little or no information on the long term effects of education and self-management advice on pain and disability outcomes. | Education and advice on prognosis and self-care (such as encouraging the patient to stay active, discourage the use of cervical collars, and appropriate support with 1 **(not 2 or more)** firm pillow are effective for relieving neck pain.  While mostly found to be effective, empirical effects are either small and effects are difficult to isolate as education and advice for neck pain are often offered in combination with other interventions including pharmacology, and or exercise. | Limited evidence from reviews show that education and advice strategies produce limited benefit for pain, function, global perceived effect, quality of life, or satisfaction in patients with cervical radiculopathy symptoms (Gross et al 2012). | ****Limited evidence** | Evidence that oral and written advice, education as part of exercise & behavioural programs are effective for reducing pain and disability and improving function in patients with acute neck pain are mostly gathered from expert opinions in guidelines and other reviews (MOM 2014; Meeus et al 2012). |
| **Shoulder pain**   - *General shoulder pain* - *Rotator cuff disorders* - *Shoulder impingement syndrome* - *Frozen shoulder/Adhesive Capsulitis* - *Acromioclavi-cular joint disorder* | No intervention/ Usual care | | Evidence reviewed across a wide range of patient prognostic profiles including acute and chronic situations, recurrent shoulder pain diagnosis, high to low baseline pain and disability | Pain  Function / disability | Long and short term | Patient education regarding physical contributory factors, and analgesics, self-help advice, on simple exercises and precautions are reported to aid the reduction of pain symptoms and gradual return to function. | Advice to avoid precipitating movements between 0 and 120^0^ for rotator cuff disorders.  Overhead activities were considered beneficial. | **Very weak evidence** | Education and self-management advice are often reported as usual complementary to other interventions. There is absence of empirical evidence and recommendations for use are largely based on expert opinions and reviews (ZGG 2004, Hazleman 2005, Mitchell 2008, Burbank et al 2008). |
| **Multisite Pain** | No intervention/ Usual care | | Chronic cases | Quality of life related outcomes | Long term | Patient educational programmes (including online courses and leaflet, information on self-care and fitness) have been shown to enhance self-efficacy and health perception (BPS 2012, Mannerkorpi & Henriksson, 2007). | n/a | ****Limited evidence** | Limited-Moderate efficacy for patients who were treated |
| **Knee Pain**   - *Overuse injuries / tendonitis* - *Patellofemoral syndrome* - *Meniscal tears; Ligament stress / strain & Soft tissue injuries* - *Knee Bursitis* - *Degenerative knee pain / Osteoarthritis* | Attention controls (Kroon et al 2014). | | Generalisable across age ranges (≥18 years) and varying pain severities. | Functional disability | Short term. | Education and patient advice suggested for supportive self-management offers marginal benefit for improvement in function (MoM 2014). | - RICE protocol (Rest, Ice, Compression and Elevation) is a core treatment offered along with self-management education and is found to be effective for the management of acute knee disorders accompanied by swelling, as well as Grade I/II MCL tears (NZGG, 2004; MoM 2014). - Weight management education and advice (for overweight or obese patients) have been suggested to aid effective management of degenerative OA (AAOS 2008; NICE 2014; Zhang et al. 2010). | * **Very weak evidence**  *** Moderate evidence for the effectiveness of RICE protocol and / or weight management (where applicable) in conjunction with education. | The effectiveness of education and self-management advice appears to be highly dependent on compliance to guidance which may not always be guaranteed due to the non-facility based nature of this treatment option.  Evidence based mostly on expert opinions or consensus in guidelines) for moderate efficacy**.** |
| **Back pain** | Usual care | | Acute and chronic LBP presentations.  The effect of educational booklets as a stand-alone intervention on pain, disability or psychological distress is not significantly different for acute and chronic low back pain presentations (Burton et al, 1999; Liddle et al, 2007; Roberts et al, 2002) | Reduction in pain and disability Improvement in function  Quality of life / work related outcomes | Long and short term | Self-management education and advice is recommended as 1st line-management of acute, non-recurrent back pain for small but clinically important effects on pain and disability in people with LBP.    WMD at short-term follow-up for pain = -3.2 points on a 0-100 scale (95% CI -5.1, -1.3) and for disability = -2.3 points (95% CI -3.7, -1.0). The long-term effects were -4.8 (95% CI -7.1, -2.5) for pain and -2.1 (95% CI -3.6, -0.6) for disability (Oliveira et al. 2012). | Group educational programmes appeared to be more effective for improving function and return to work than individual programmes for acute back pains but additional interventions may be required for more chronic conditions (Engers et al, 2008; Heymans et al, 2004; 2006).  Advice for bed rest is not effective for reducing pain and improving function in both acute and chronic LBP presentations (Dahm et al. 2010; Hagen et al, 2004; MOM 2014; NICE 2009).  Advice to stay active lead to small improvements in pain relief (SMD 0.22 (95% CI: 0.02 to 0.41) and functional status (SMD 0.29 (95% CI: 0.09 to 0.49) (Dahm et al.2010). | *****Moderate evidence**  **Small effects** |  |

*Very weak evidence: Expert opinions or consensus in guidelines only / Absence of evidence in a single systematic review.

** Limited evidence: little empirical evidence from systematic reviews/evidence-based guidelines AND when there were small, inconsistent, or non-significant treatment effect sizes.

*** Moderate evidence: little empirical evidence from systematic reviews/evidence-based guidelines (as in limited evidence) but showing a medium to large treatment effect OR in the presence of strong empirical evidence from high quality systematic reviews, but with small or inconsistent treatment effect sizes across systematic reviews.

**** Strong evidence: strong empirical evidence from high quality systematic reviews and evidence based clinical guidelines AND medium or large effect sizes.
