# Supplementary material for: Effective treatment options for musculoskeletal pain in primary care: A systematic overview of current evidence
Source: PLoS One. 2017 Jun 22;12(6):e0178621. doi: 10.1371/journal.pone.0178621 (PMC5480856; doi:10.1371/journal.pone.0178621)
Supplement: S2 Table — (DOCX) [file pone.0178621.s004.docx]

|  | | **Compendium of evidence on analysis of effectiveness of exercise across regional musculoskeletal pain presentations** | | | | | | | |
| --- | --- | --- | --- | --- | --- | --- | --- | --- | --- |
| **Regional pain**  *(Sub-diagnosis)* | **Comparison (s)** | | **Specific patient profiles/ mediating risk factors**  *(e.g., pain severity @baseline; pain duration; previous pain episodes; age; movement restriction; baseline disability)* | **Outcomes**  *Pain*  *Functional Disability*  *& other 2 ^0^ Outcomes* | **Long term / short term** | **Results /Effect size** | **Specific Diagnostic considerations** | **Grade of evidence** | **Comments / summary of evidence** |
| **Neck Pain**   - *Whiplash injury/ Whiplash associated disorders (WAD)* - *Non-specific neck pain* - *Acute torticollis* - *Cervical radiculo-pathy* | sham or no treatments | | acute and chronic neck pain patients | Pain  Function | Long term / short term | Exercises are effective for improving function and reducing pain and disability in patients with acute and chronic neck pain (Hurwitz et al. 2008; MOM 2014; Teasell et al. 2010).  Exercise interventions with particular emphasis on functional rehabilitation appear to be more effective than interventions that did not have such a focus (Hurwitz et al, 2008). | n/a | ****Limited evidence**  **Medium effects** | Exercise is effective for the management of all neck pain diagnosis but the evidence on the relative effectiveness of different exercise regimens when compared with each other is inconclusive. |
| **Shoulder pain**   - *General shoulder pain* - *Rotator cuff disorders* - *Shoulder impingement syndrome* - *Frozen shoulder/Adhesive Capsulitis* - *Acromioclavi-cular joint disorder* | Surgical management;  Other conservative treatments (e.g., Corticosteroid injections, analgesics);  Placebo/Sham treatment/no treatment. | | Considered for wide range pain severity @baseline; pain duration; recurrent shoulder pain episodes, and functional disability levels. | Pain  Function  Quality of life  Range of movement | In the short term or acute phase exercise alone appears less effective for improving pain and function compared with intra-articular corticosteroid injections (Cleland and Durall 2002, Green et al 2003, Murphy and Carr 2010, Littlewood 2012). | Physio-led exercise has significant positive effects on recovery from pain: RR 7.74 (1.97, 30.32), function: RR 1.53 (0.98, 2.39), and ROM 0.33 (0.11, 0.96) in the short term and long term compared with other non-surgical interventions (Marinko et al. 2011; Green et al 2003).  Evidence of significant and sustained (up to 2.5 year follow up) benefit for exercise compared with placebo in terms of function (RR 7.74, 95% CI 1.97 to 30.32), with additional benefit from exercise plus mobilization (Green et al, 2003; Grant et al, 2004).  Compared with surgical procedure, placebo or no treatment, good level evidence for beneficial effects of exercises on pain, function, and QoL in the short-term (Hanratty et al 2012, Desmeules et al 2003, Kuhn 2009 & Kromer 2009). | On strength of the rotator cuff, exercise has a small positive effect in the short term (SMD -0.46 (-0.76, 0.16); p = 0.003) which is also sustained on long-term function (SMD -0.31 (-0.57, 0.04); p = 0.02) (Hanratty et al 2012). | ******Strong evidence**  **Large effects** | Especially in the very acute/ early phase of shoulder pain diagnosis, exercise generally well tolerated and effective post 4-6 weeks of pain management with analgesics / corticosteroids injections (if indicated). |
| **Multisite Pain** | Usual Care | | Chronic | Pain  Psychosocial pain related outcomes e.g., anxiety. |  | Both group therapy and individual therapy effective for relieving symptoms due to multi-site pain (Mannerkorpi & Henriksson, 2007; BPS 2012).  Aerobic exercises effects improvement in global well-being (SMD 0.49, 95% CI: 0.23 to 0.75) and physical function (SMD 0.66, 95% CI: 0.41 to 0.92) and pain (SMD 0.65, 95% CI: -0.09 to 1.39) of adults with widespread pain including fibromyalgia (Busch et al 2007; Hauser et al 2010). | n/a | ****** Strong evidence**  **Medium effects** | Though exercise is effective in the management of multi-site pain, there is no strong recommendation for a specific/ standard exercise regime. |
| **Knee Pain**   - *Overuse injuries / tendonitis* - *Patellofemoral syndrome* - *Meniscal tears; Ligament stress / strain & Soft tissue injuries* - *Knee Bursitis* - *Degenerative knee pain / Osteoarthritis* | Usual care  Surgery  Other conservative treatments | | acute and chronic presentations | Pain  Function | Long & Short term | With a general focus on muscle rehabilitation, exercise and proprioceptive training to retain protection and stability of the joint is effective for the reduction of pain and improving function across knee pain presentations (Bartels et al. 2007 & Fransen et al 2008; Hochberg et al 2012, MoM 2014; NZGG 2004; NICE 2014, Zhang et al 2010).  For the management of patellofemoral knee pain (van der Heijden et al. 2015), pooled data for pain during activity favoured exercise therapy compared to controls (MD -1.46, 95%, CI -2.39 to -0.54 (scale 0 to 10) as well functional ability (SMD 1.10, 95% CI 0.58 to 1.63) in the short term. | No evidence for the effectiveness of exercise on the management of knee bursitis  For overuse injuries and ligamental stress presentations, physiotherapy exercises to include eccentric stretching of tendons/ ligaments (MoM, 2014). | **** *Moderate evidence**  **Medium effects** | There is little evidence in favour of any particular exercise program/ regime (e.g. aquatic, aerobic, and resistance exercises, stretching and tai chi) over the other.  Adherence to exercise therapy was lower for studies presenting small effect sizes. |
| **Back pain** | No intervention  Surgery  Other conservative treatment options. | | acute and chronic LBP | Pain  Functional disability  Work related outcomes  Quality of life. | Long & Short term | - Exercise has been proven effective for the reduction of pain and improving function in acute and chronic LBP (MoM 2014; NICE 2009; Hayden et al, 2005). NNT for improvement or benefit at 3 months: 8.2 (95% CI 4.9 to 25.0) and 6.6 (95% CI 4.1 to 16.6) (Froud et al, 2009). - There is significant effect in favour of exercise on work disability in the long term (odds ratio (OR) = 0.66, 95% CI 0.48-0.92) but not in the short (OR = 0.80, 95% CI 0.51-1.25) and intermediate term (OR = 0.78, 95% CI 0.45-1.34) (Oesch et al. 2010). - Post-treatment exercises are effective for reducing rate of recurrences at one year (Rate Ratio 0.50; 95% CI 0.34 to 0.73) compared to no intervention. (Choi et al., 2010). | †There is little empirical evidence in favour of any particular exercise program/ regime/ mode of delivery either structured individual or group over the other (Aladro-Gonzalvo et al. 2012; Hayden et al, 2005; Kuczynski et al. 2012; Macedo et al, 2009; MoM 2012; NICE 2009; Pereira et al.2012; Rasmussen-Barr et al, 2009; Waller et al, 2009; Wang et al. 2012; Wells et al.2013). | ****** Strong evidence**  **Medium/large effects** |  |

* Very weak evidence: Expert opinions or consensus in guidelines only / Absence of evidence in a single systematic review.

** Limited evidence: little empirical evidence from systematic reviews/evidence-based guidelines AND when there were small, inconsistent, or non-significant treatment effect sizes.

*** Moderate evidence: little empirical evidence from systematic reviews/evidence-based guidelines (as in 2) but showing a medium to large treatment effect OR in the presence of strong empirical evidence from high quality systematic reviews, but with small or inconsistent treatment effect sizes across systematic reviews.

**** Strong evidence: strong empirical evidence from high quality systematic reviews and evidence based clinical guidelines AND medium or large effect sizes.
