# Supplementary material for: Effective treatment options for musculoskeletal pain in primary care: A systematic overview of current evidence
Source: PLoS One. 2017 Jun 22;12(6):e0178621. doi: 10.1371/journal.pone.0178621 (PMC5480856; doi:10.1371/journal.pone.0178621)
Supplement: S3 Table — (DOCX) [file pone.0178621.s005.docx]

|  | | **Compendium of evidence on analysis of effectiveness of manual therapy and massage across regional musculoskeletal pain presentations** | | | | | | | |
| --- | --- | --- | --- | --- | --- | --- | --- | --- | --- |
| **Regional pain**  *(Sub-diagnosis)* | **Comparison (s)** | | **Specific patient profiles/ mediating risk factors**  *(e.g., pain severity @baseline; pain duration; previous pain episodes; age; movement restriction; baseline disability)* | **Outcomes**  *Pain*  *Functional Disability*  *& other 2 ^0^ Outcomes* | **Long term / short term** | **Results /Effect size** | **Specific Diagnostic/Prognostic considerations** | **Grade of evidence** | **Comments / summary of evidence** |
| **Neck Pain**   - *Whiplash injury/ Whiplash associated disorders (WAD)* - *Non-specific neck pain* - *Acute torticollis* - *Cervical radiculo-pathy.* | Exercise therapy alone/usual care  Placebos | | Range from low to severe neck pain, and disability. | range of motion  function  Pain | Manipulation and mobilization are effective for immediate and or short-term improvement in pain, range of motion and function in both acute and chronic neck pain patients (Hurwitz et al. 2008; MOM 2014; Teasell et al. 2010). | One to four sessions of cervical Manipulation produced short- term relief compared to controls (SMD pooled -0.90 (95%CI: -1.78 to -0.02) in subacute/chronic neck pain patients (Gross et al. 2012).  Thoracic Manipulation led to significant pain reduction (NNT 7; 46.6%) and increased function (NNT 5; 40.6%) in acute neck pain patients.  For chronic neck pain patients, a single session of thoracic manipulation led to immediate pain reduction (NNT 5, 29%) compared to placebo (Gross et al. 2012). | Evidence not applicable to acute torticollis and cervical radiculopathy.  As an adjunct treatment, therapeutic massage is an effective intervention for immediate-post treatment reduction in symptoms for both sub-acute and chronic mechanical neck disorders (Patel et al. 2012, Ottawa Panel 2012). | ****Limited evidence**  **Small to Medium effects** | Usually in combination with exercise, manual therapy, when indicated, is an effective intervention for reducing pain and improving function in neck pain presentations.  There is a general lack of specificity about the optimum technique, number of sessions or professional administering manual therapy. |
| **Shoulder pain**   - *General shoulder pain* - *Rotator cuff disorders* - *Shoulder impingement syndrome* - *Frozen shoulder/Adhesive Capsulitis* - *Acromioclavi-cular joint disorder* | Manual therapy combined with or without exercise  Manual therapy combined with or without multimodal therapy (including surgery).  Pharmacological therapy  placebo, / no intervention | | Generalisable across different shoulder pain morbidity profiles | range of motion  function  Pain | Long and short term | Where strongly indicated, and professionally administered, manual therapy offers some benefit on improving range of motion and functional disability in shoulder pain presentations. (Camarinos & Marinko 2009, Bokarius & Bokarius 2010, Brudvig et al 2011, Brantingham et al 2011, Desmeules et al 2003; Ho et al 2009). | Evidence not applicable to acromioclavicular joint disorder.  Considerations for supplementing manual therapy with other pain-relieving modalities e.g corticosteroid injections for improve pain in the short term | **** Limited evidence**  **Small effects** | The minimal added benefits of manual therapy are accrued usually in combination with other treatment modalities, and mostly in non-acute presentations. |
| **Back pain** | Manual therapy combined with exercise or analgesics.  Manual therapy combined with or without multimodal therapy (including surgery). | | - Evidence for sub-group specific targeted intervention improves efficacy of manual therapy (Slater et al. 2012) | function  Pain | Short-term | - Manual therapies/spinal manipulations alone or in combination with exercise or analgesia are effective for reducing pain & disability (Bronfort et al. 2009; Ernst and Harkness, 2001; Goertz et al. 2012; NICE 2009). - Short-term effect on chronic pain relief (MD: -4.16, 95% CI -6.97 to -1.36) and functional status (SMD: -0.22, 95% CI -0.36 to -0.07) compared to other interventions. (Rubinstein et al. 2011; 2012). | For regimes that were professionally administered, the risk of major adverse events is low, but about 50% of patients may experience minor to moderate adverse events such as muscle soreness, aching and headache. Most adverse effects (mean 67%, range 55-83%) were also resolved within 24 hours post treatment. The relative risks are lower (RR 0.05, CI 95% 0.01–0.20) compared with pharmacological interventions but higher (RR1.91, CI 95%1.39–2.64) compared with usual care (Carnes et al 2010).  As a stand-alone intervention, the positive effects of massage on pain & disability is short-lived and not cost-effective (Furlan et al, 2008). | ****Limited evidence**  **Small – medium effects.** | Low-moderate evidence of clinical efficacy of spinal manipulative therapy may not justify cost effectiveness. Hence, recommendations for use where strongly indicated / improved with sub-group targeting on the basis of prognostic factors. |

*Very weak evidence: Expert opinions or consensus in guidelines only / Absence of evidence in a single systematic review.

** Limited evidence: little empirical evidence from systematic reviews/evidence-based guidelines AND when there were small, inconsistent, or non-significant treatment effect sizes.

*** Moderate evidence: little empirical evidence from systematic reviews/evidence-based guidelines (as in limited evidence) but showing a medium to large treatment effect OR in the presence of strong empirical evidence from high quality systematic reviews, but with small or inconsistent treatment effect sizes across systematic reviews.

**** Strong evidence: strong empirical evidence from high quality systematic reviews and evidence based clinical guidelines AND medium or large effect sizes.

***Evidence not applicable to knee and multi-site pain**
