# Supplementary material for: Effective treatment options for musculoskeletal pain in primary care: A systematic overview of current evidence
Source: PLoS One. 2017 Jun 22;12(6):e0178621. doi: 10.1371/journal.pone.0178621 (PMC5480856; doi:10.1371/journal.pone.0178621)
Supplement: S4 Table — (DOCX) [file pone.0178621.s006.docx]

|  | | **Compendium of evidence on analysis of effectiveness of pharmacological therapy across regional musculoskeletal pain presentations** | | | | | | | |
| --- | --- | --- | --- | --- | --- | --- | --- | --- | --- |
| **Regional pain**  *(Sub-diagnosis)* | **Comparison (s)** | | **Specific patient profiles/ mediating risk factors**  *(e.g., pain severity @baseline; pain duration; previous pain episodes; age; movement restriction; baseline disability)* | **Outcomes**  *Pain*  *Functional Disability*  *& other 2 ^0^ Outcomes* | **Long term/ short term** | **Results /Effect size** | **Specific Diagnostic considerations** | **Grade of evidence** | **Comments / summary of evidence** |
| **Neck Pain**   - *Whiplash injury/ Whiplash associated disorders (WAD)* - *Non-specific neck pain* - *Acute torticollis* - *Cervical radiculo-pathy.* | Placebo &  Other Pharmacolo-gical agents  Corticosteroid injections vs exercise therapy/ usual care | | Evidence across varying neck pain morbidity profiles | Pain | Long & Short term | **Analgesics**  Paracetamol and/or ibuprofen are effective for pain relief. Codeine may be added to regular paracetamol but combination products e.g. cocodamol, are not recommended (MOM 2014).  **Injections**  Fluoroscopically guided injections; depot corticosteroids and botulinum toxin injections are effective for short term relief of pain for specific, sub-acute and chronic neck pain patients but therapeutic benefits are not sustained (Institute for Clinical Systems Improvement - ICSI, 2011, Langevin et al. 2011).  Sterile water, saline and botulinum toxin injections are not very effective in the treatment of WAD (Teasell et al. 2010).    Epidural steroid injections are effective for short-term pain relief for patients with cervical radiculopathy from degenerative disorders (Bono et al 2011; Diwan et al 2012; Peloso et al. 2007). | In chronic cases of radiculopathy, low-dose amitriptyline and anti-epileptics may be beneficial if there has been no response to conventional analgesics (paracetamol, non-steroidal anti-inflammatory drugs, or opioids) (MOM 2014).  Addition of steroid to local anaesthetic does not confer improved relief in symptoms in the long term (Bono et al 2011; Diwan et al 2012). | ****Limited evidence**  **Small to Medium effects** | Pharmacological therapies appear effective for pain relief across all neck pain diagnosis but the beneficial effects are small and un-sustained beyond short term applications.  *Evidence regarding corticosteroid use for neck pain is not applicable to acute torticollis presentations. |
| **Shoulder pain**   - *General shoulder pain* - *Rotator cuff disorders* - *Shoulder impingement syndrome* - *Frozen shoulder/Adhesive Capsulitis* - *Acromioclavi-cular joint disorder* | Placebo  Other Pharmacolo-gical agents  Corticosteroid injections/ usual care | | Acute & Sub acute presentations | Pain | Short term | **Analgesics**  NSAIDs and/ or analgesics equally effective as first line management for short term symptom relief together with advice for gentle stretching and strengthening exercises (Burbank et al 2008; Hazleman 2005; Mitchell, 2008;MoM 2014; NZGG 2004; van der Sande 2013).    NSAIDs & analgesics are more effective than placebo for pain & function and as good as corticosteroids in acute phase (Johansson et al, 2002; Min et al 2013).  **Injections**  Beneficial effects of corticosteroid injections for improving pain in the short term: 3-9 months (Buchbinder et al 2003, Arroll & Smith 2005; Murphy and Carr 2010; Shah & Lewis, 2007). May be considered for second line management in the event of failed treatment with analgesics.   - Short-lasting effects, so corticosteroid injection may possibly be used as baseline therapy prior to or alongside physiotherapy. As a stand-alone treatment, corticosteroid injections not found to be necessarily better than physiotherapy in the long term. - Multiple injections up to 3 seem more beneficial than a single dose. More than 4 doses do not confer significant additional benefits. High dose corticosteroids may be more effective than low dose. | Based on expert reviews: Acromioclavicular joint problems without dislocation usually resolve with rest and simple analgesia (Mitchell, 2008). | *****Moderate evidence**  **Medium effects** | Effect may be small /medium and not well-maintained; no significant benefit in terms of pain, ROM and function at 12 months for injection.  Small sample sizes, variable methodological quality, and heterogeneity limit conclusions on effectiveness. |
| **Multisite Pain** | Placebo  Varying Pharmacological agents | | Chronic | Pain  Pain related psychological outcomes | Long term | Step-wise analgesia (including weak opioid, tramadol, topical lidocaine) have been found effective for pain relief (BPS 2012; Stanos & Galluzzi, 2013).  Other Pharmacological agents (e.g., amitriptyline or nortriptyline, fluoxetine, duloxetine, Gabapentin or pregabalin) may be prescribed on needs basis (BPS 2012; Choy et al 2010; Moore et al 2014; Siler et al 2011; Carville et al 2008; Hauser et al 2012). | Pregabalin and gabapentin are modestly effective leading to about 35% of patients achieving 50% reduction in degree of pain with gabapentin, compared with 21% for placebo, (Moore et al 2014; Siler et al 2011; Carville et al 2008; Hauser et al 2012; BPS 2012).  However, long term safety and efficacy are unknown (Siler et al 2011). | *****Moderate evidence**  **Moderate effects** |  |
| **Knee Pain**   - *Overuse injuries / tendonitis* - *Patellofemoral syndrome* - *Meniscal tears; Ligament stress / strain & Soft tissue injuries* - *Knee Bursitis* - *Degenerative knee pain / Osteoarthritis* | Placebo  Usual care | | Low to moderate /severe pain  Acute and chronic presentations | Pain  Functional disability | Short term | **Analgesics**  Topical or oral NSAIDs offer safe short term benefit for pain and function (Heintjes et al. 2004; MOM 2014, NZGG 2004).  Paracetamol: recommended as first line pharmacological management of meniscal injuries considering also its cost-effectiveness and ratio of benefit to harm (NZGG 2004).  **Injections**  Corticosteroid injections are effective in the short term (1-4 weeks) for relieving moderate to severe pain compared to placebo ((RR: 3.11 (95% CI 1.61 to 6.01); (WMD -21.91; 95% CI -29.93 to -13.89); NNT of 3 to 4)). However, the beneficial effect of intra-articular injections on functional improvement is less demonstrated. (AAOS, 2008; Belamy et al 2006, Heintjes et al. 2004; NICE, 2008).  The onset and size of effect of corticosteroids was similar to, but was less durable (from 4-5 weeks) than with hyaluronan injections (Belamy et al 2006).  Hyaluronic injections have been found to be effective for reducing OA pain up to 8-12 weeks but the evidence for its use over other treatment options is inconclusive considering small effect size and cost-effectiveness (AAOS, 2008; Bannuru et al. 2011; California Technology Assessment Forum 2012; Hochberg et al 2012, NICE 2014). | *For knee bursitis:*   - NSAIDs may be beneficial for treating a persistent effusion that is refractory to the 'RICE protocol' 48 hours after the initial injury (NZGG 2004). Anti-inflammatory medication and /or antibiotics are recommended in case of suspected infection (MoM 2014).   *For degenerative knee pain*:   - Regular dosage of paracetamol is effective though effects is sometimes small compared to NSAIDs (Cepeda et al 2006; NICE 2014, Expert opinion). - Oral NSAIDs)/COX-2 inhibitors have similar analgesic effects and should be used at the lowest effective dose for the shortest possible period of time (NICE 2014, Zhang et al 2008). - NSAIDs known to be more effective than acetaminophen (Towheed et al 2006). - Topical NSAIDs: American College of Rheumatology and Recent Cochrane review found topical diclofenac to be equivalent to (or better than) oral NSAIDs (Derry et al 2012). Topical NSAIDs also have lower incidence of systemic adverse events compared with oral NSAIDs but can be can be associated with local adverse events such as dry skin, erythema, and itching (Derry et al 2012). - Non-tramadol opiods has harms outweighing is small clinical effect (Nuesch et al 2009). Opioids should be prescribed at a low starting dose, only for patients in whom other analgesia is contraindicated or ineffective (Hochberg et al. 2012; NICE, 2008). - The evidence for the efficacy of capsaicin is inconclusive (NICE 2014, American College of Rheumatology, 2008). - Nutraceuticals (chondroitin and glucosamine) have not been shown to have significant beneficial effect on joint pain, or joint space narrowing and function compared with placebo (AAOS, 2008; Hochberg et al 2012; NICE 2014, Rutjes et al. 2009 & Toehhed et al. 2005; Wandel et al. 2010). - Diacerin has minimal efficacy for symptomatic relief (Fidelix et al 2014). - Doxycyline was considered not effective as benefit does not outweigh the harm (da Costa et al 2012). | ****** Strong-moderate evidence**  **Moderate effects** |  |
| **Back pain** | placebo | | Acute /Chronic  Mild to severe pain presentations | Pain  Function | The evidence for pain relief across the common pharmacological ineterventions is valid mostly in the short term (Kuijpers et al. 2011). | - NSAIDs are more effective than placebo for short-term pain relief in either acute or chronic (non-sciatica; WMD of –12.40 (95% CI –15.53 to –9.26, p < 0.00001)) LBP without sciatica. - Weak opioids have been found effective for pain relief compared to NSAIDs but the effect sizes are small and not maintained in the long-term after treatments/dosages were stopped or reduced. The risks of adverse effects and dependence are also high (Deshpande et al, 2007; Martell et al, 2007; NICE 2009). - Tricyclic antidepressants (TCAs) and selective serotonin reuptake inhibitors (SSRIs) do not confer beneficial effects on pain, function, anxiety or depression associated with LBP (NICE 2009; Urquhart et al, 2008). - Combination therapy with (non-benzodiazepine) muscle relaxants and analgesic or NSAIDs appears more effective (small effects, not statistically significant) than analgesic or NSAID alone for short-term pain relief and reduction of muscle spasm in acute LBP. However, with increased rate/risk of adverse effects (van Tulder et al, 2003). - Combined therapy generally shows efficacy but increased side effects (Romano et al. 2012) - Glucosamine food supplements have no effect on LBP (Sodha et al 2013). | Equivocal evidence on the effectiveness of topical over oral NSAID (vice versa) for acute LBP.  Compared to paracetamol, the evidence for better treatment outcome with NSAIDs is weak for chronic LBP  NSAIDs and coxibs appear equally effective (Roelofs et al, 2008).  Neuropathic pain treatment (e.g. amitriptyline, pregabalin) may be considered in the presence of radiculopathy (MoM 2014).  Botulinum neurotoxin injections do not lead to clinically significant improvement in pain, and or function when compared to acupuncture or steroid injections. (Waseem et al. 2011).  Recent RCT evidence finds oral antibiotics effective for improving pain and function in chronic LBP patients with previous history of disc herniation (Albert et al 2013). | *****Moderate evidence**  **Medium effects** | - Guidelines (MoM 2012; NICE; 2008; 2014) recommends paracetamol for pain relief but the validity of the recommendation has been questioned by reviews which found no evidence for current practice (Davies et al 2008). |

* Very weak evidence: Expert opinions or consensus in guidelines only / Absence of evidence in a single systematic review.

** Limited evidence: little empirical evidence from systematic reviews/evidence-based guidelines AND when there were small, inconsistent, or non-significant treatment effect sizes.

*** Moderate evidence: little empirical evidence from systematic reviews/evidence-based guidelines (as in 2) but showing a medium to large treatment effect OR in the presence of strong empirical evidence from high quality systematic reviews, but with small or inconsistent treatment effect sizes across systematic reviews.

**** Strong evidence: strong empirical evidence from high quality systematic reviews and evidence based clinical guidelines AND medium or large effect sizes.
