# Supplementary material for: Effective treatment options for musculoskeletal pain in primary care: A systematic overview of current evidence
Source: PLoS One. 2017 Jun 22;12(6):e0178621. doi: 10.1371/journal.pone.0178621 (PMC5480856; doi:10.1371/journal.pone.0178621)
Supplement: S5 Table — (DOCX) [file pone.0178621.s007.docx]

|  | | **Compendium of evidence on analysis of effectiveness of other, complementary & alternative therapy across regional musculoskeletal pain presentations** | | | | | | | |
| --- | --- | --- | --- | --- | --- | --- | --- | --- | --- |
| **Regional pain**  *(Sub-diagnosis)* | **Comparison (s)** | | **Specific patient profiles/ mediating risk factors**  *(e.g., pain severity @baseline; pain duration; previous pain episodes; age; movement restriction; baseline disability)* | **Outcomes**  *Pain*  *Functional Disability*  *& other 2 ^0^ Outcomes* | **Long term / short term** | **Results /Effect size** | **Specific Diagnostic considerations** | **Grade of evidence** | **Comments / summary of evidence** |
| **Neck Pain**   - *Whiplash injury/ Whiplash associated disorders (WAD)* - *Non-specific neck pain* - *Acute torticollis* - *Cervical radiculo-pathy.* | Placebo/ usual care | | Across spectrum of long to short pain duration and severity. | *Pain*  *Functional Disability* | Long term & short term | Acupuncture is effective for short term relief of neck pain (Trinh et al 2006).  Expert consensus considers postural adjustments (such as sleeping on a low firm pillow) as effective management options in conjunction with education advice and analgesics (MOM 2014). | Collars and tractions tend to induce rest and inactivity hence prolongs disability (Carroll et al. 2009; Hurwitz et al. 2008; MOM 2014; Teasell et al. 2010).  Traction and acupuncture offer limited benefits for relieving symptoms due to cervical radiculopathy (Trinh et al. 2006).  Intermittent heat or cold packs are effective for relieving pain and spasm due to torticollis. | ****Limited evidence**  **Small effects** | Inconclusive evidence for the beneficial effect of Ultrasound/TENS, Laser/Acupuncture/Pulsed EMF treatment on neck pain symptoms(Carroll et al. 2009; Hurwitz et al. 2008; Kadhim-Saleh et al 2013; MOM 2014; Teasell et al. 2010). |
| **Shoulder pain**   - *General shoulder pain* - *Rotator cuff disorders* - *Shoulder impingement syndrome* - *Frozen shoulder/Adhesive Capsulitis* - *Acromioclavi-cular joint disorder* | Placebo/Usual care/ other treatment options | | Across spectrum of long to short pain duration and severity. | *Pain*  *Functional Disability* | Short & long term | Ultrasound , TENS and acupuncture has some benefits in reducing pain and improving function (Faber et al, 2006; Green et al, 2003; 2005; Grant et al, 2004, Johansson 2002; Kromer 2009; Maund et al 2012; NZGG 2004; Robertson 2001; Vickers et al 2012). | No differences in effects of TENS compared to ultrasound for improving pain and function (Johansson 2002).  In conjunction with exercise, acupuncture appears better than ultrasound but has comparable efficacy with corticosteroid injections and exercise (Johansson et al. 2005; 2011).  Assistive devices, e.g. supports/braces have been found to be effective for patients with biomechanical joint pain or instability as part of physio- treatment (MoM 2014). | **** Limited evidence**  **Small effects** | Irrespective of acuteness or chronicity of shoulder pain presentations, ultrasound, laser, TENS, acupuncture as stand-alone treatment do not confer added benefits over placebo and other treatment options. |
| **Multisite Pain** | Usual care | | Chronic pain presentations | Pain  Function  Quality of life related outcomes. | Short term | In combination with massage, acupuncture led to minimal symptom relief for multisite pain patients but the effects were not sustained (Mannerkorpi & Henriksson, 2007). | N/A | ***Very weak evidence** |  |
| **Knee Pain**   - *Overuse injuries / tendonitis* - *Patellofemoral syndrome* - *Meniscal tears; Ligament stress / strain & Soft tissue injuries* - *Knee Bursitis* - *Degenerative knee pain / Osteoarthritis* | Usual Care | | Across acute/chronic to single and recurrent episodes of knee pain | Pain  Function | Long term/short term | Evidence neither support or reject the effectiveness of orthotics, taping techniques and other complementary therapies as stand-alone treatment options in reducing pain. (AAOS, 2008; D'hondt et al 2002; Hochberg et al 2012, Manheimer et al 2010; NHMRC, 2009; & NICE 2014).  Ultrasound, laser, TENS, biofeedback, electromagnetic fields, neuromuscular electrical stimulation (NMES) may confer added benefits to rehabilitation and or surgical management (NZGG 2003). However, effects are small and are only supported by weak evidence (NHMRC, 2009; NICE 2014; Rutjes et al. 2009a & b). | - Marginal pain relief (over the short term) presents with no clear advantage of foot orthoses over simple insoles or physiotherapy for patello-femoral pain (Hossain et al 2011). - On the basis of expert opinions, guidelines recommend hinged brace for 4-6 weeks in grade II and III collateral ligament injuries as effective rehabilitative procedure (MoM 2012; NZGG 2003). - The evidence for the efficacy of acupuncture in the treatment of Knee OA is inconclusive due to inconsistencies in method of application, results, and limited data on its clinical and cost effectiveness (AAOS, 2008; Hochberg et al 2012, Manheimer et al 2010; NHMRC, 2009; NICE 2014). - For degenerative knee pain, Ice massage confer some benefit for ROM, function and knee strength while cold packs relieved oedema. Hot packs had no beneficial effect on oedema and Ice packs have no significant effect on pain reduction (Brosseau et al 2003). - There are uncertainties in the balance of benefits vs. harms with topical herbal treatments (Cameron et al 2013). - Electromagnetic field (EMF) therapy may provide moderate pain relief in OA but its effects on function and quality of life is uncertain.(MD 15.10, 95% CI 9.08 to 21.13; absolute improvement 15%) after 4 to 26 weeks’ treatment compared with placebo. - Recent Cochrane evidence shows that there was no statistically significant difference in adverse event of EMF (RR 1.17, 95% CI 0.72 to 1.92) compared with placebo (li et al 2013). | ***Very weak evidence** | low effectiveness when used as stand-alone treatment  Evidence suggest multimodal therapy including exercise and or pharmacological agents for effective pain relief and increasing function   - **NICE 2014 advise against acupuncture.** |
| **Back pain** | Usual Care/ other active complementary alternatives. | | Acute/Chronic | Pain  Function | Long & Short term. | - Acupuncture is found effective for short term pain relief but effects are very small, not evident for functional outcomes and were not maintained at longer-term follow up (Furlan et al, 2005; Hutchinson et al. 2012; Lee et al. 2013; NICE 2009). - TENS may be no more effective for reducing pain than placebo in chronic LBP presentations (Dubinski et al. 2010, Middlekoop et al. 2011). - Ultrasound and shockwave therapy not found effective for acute/chronic LBP (Seco et al.2011). - In combination with exercise, superficial heat therapy may reduce pain and improve function in acute and sub-acute low-back pain presentations. The evidence for cold therapy is even more limited (French et al, 2006) - Weak evidence of limited effectiveness precludes conclusion on yoga, osteopathic and chiropractic interventions (Orrock &Myers 2013; Parkinson et.al 2012; Posadzki & Ernest 2011; Walker et al. 2010). |  | ****Limited evidence**  **Small/medium effects.** | Current knowledge on effectiveness of complementary therapies for low back pain is based on considerable amount of evidence without convincing effect sizes. |

*Very weak evidence: Expert opinions or consensus in guidelines only / Absence of evidence in a single systematic review.

** Limited evidence: little empirical evidence from systematic reviews/evidence-based guidelines AND when there were small, inconsistent, or non-significant treatment effect sizes.

*** Moderate evidence: little empirical evidence from systematic reviews/evidence-based guidelines (as in limited evidence) but showing a medium to large treatment effect OR in the presence of strong empirical evidence from high quality systematic reviews, but with small or inconsistent treatment effect sizes across systematic reviews.

**** Strong evidence: strong empirical evidence from high quality systematic reviews and evidence based clinical guidelines AND medium or large effect sizes.
