# Supplementary material for: Effective treatment options for musculoskeletal pain in primary care: A systematic overview of current evidence
Source: PLoS One. 2017 Jun 22;12(6):e0178621. doi: 10.1371/journal.pone.0178621 (PMC5480856; doi:10.1371/journal.pone.0178621)
Supplement: S6 Table — (DOCX) [file pone.0178621.s008.docx]

|  | | **Compendium of evidence on analysis of effectiveness of psychosocial interventions across regional musculoskeletal pain presentations** | | | | | | | |
| --- | --- | --- | --- | --- | --- | --- | --- | --- | --- |
| **Regional pain**  *(Sub-diagnosis)* | **Comparison (s)** | | **Specific patient profiles/ mediating risk factors**  *(e.g., pain severity @baseline; pain duration; previous pain episodes; age; movement restriction; baseline disability)* | **Outcomes**  *Pain*  *Functional Disability*  *& other 2 ^0^ Outcomes* | **Long term / short term** | **Results /Effect size** | **Specific Diagnostic considerations** | **Grade of evidence** | **Comments / summary of evidence** |
| **Neck Pain**   - *Whiplash injury/ Whiplash associated disorders (WAD)* - *Non-specific neck pain* - *Acute torticollis* - *Cervical radiculo-pathy.* | Usual care | | Moderate to severe neck pain  Chronic / recurrent episodes.  Considerable disability on baseline assessments. | Pain  Quality of life | Long & Short term | Consideration for referral to Occupational Health or psychologist are effective for the management of neck pain when psychological factors, medico-legal issues, family dynamics or work related issues are identified. (Carroll et al. 2009; MOM 2014). | In chronic WAD, biopsychosocial interventions are critical to reducing disability and effective pain management (Carroll et al. 2009; MOM 2014). | ****** Strong evidence**  **Moderate effectiveness** |  |
| **Shoulder pain**   - *General shoulder pain* - *Rotator cuff disorders* - *Shoulder impingement syndrome* - *Frozen shoulder/Adhesive Capsulitis* - *Acromioclavi-cular joint disorder* | Usual care | | Non-specific | Pain  Quality of life  Function | Long & Short term | Psychosocial interventions lead to some improvement in functions (Bruign et al.2007; Geraets et al. 2005; Karjalainen et al. 2003). Paucity of empirical evidence limits recommendations on overall effectiveness in clinical settings. | n/a | **Limited evidence**  **Small effects** | Efficacy of bio-psychosocial interventions appears modulated by different factors such as : the professional giving treatment (Karjalainen et al. 2003; Briugn et al. 2007); settings (i.e group vs. individual, Bruign et al.2007; Geraets et al. 2005) and phase of the shoulder pain (i.e. acute vs. chronic, Geraets et al. 2005). |
| **Multisite Pain** | Usual care | | Moderate to severe pain  Chronic / recurrent episodes.  Considerable disability on baseline assessments.  Considered across different age landscape >18 years. | Quality of life  Function  Pain | Long & Short term | Multidisciplinary biopsychosocial rehabilitation (with or without vocational rehabilitation) have been found effective, if psychosocial barriers to good prognosis (e.g., distress, disability, drug escalation & dependency) are identified, (Davies et al., 2008, NICE, 2009). | Multisite pain  Chronic widespread pain  Fibromyalgia | *****Moderate evidence**  **Medium effects** | Effective management with psychosocial intervention hinged on successful baseline assessment and early identification of poor prognostic indicators. |
| **Knee Pain**   - *Overuse injuries / tendonitis* - *Patellofemoral syndrome* - *Meniscal tears; Ligament stress / strain & Soft tissue injuries* - *Knee Bursitis* - *Degenerative knee pain / Osteoarthritis* | Usual care | | Non specific | Pain  psychological, physical, and biological functioning | Non specific | Psychosocial interventions including cognitive-behavioural therapy (CBT)/pain-coping skills training, biofeedback, stress management, emotional disclosure, hypnosis and psychodynamic therapy had some positive effects on pain and pain-related psychological outcomes (Dixon et al, 2007). | Osteoarthritis | **Limited Evidence** | Relatively small effect sizes, weak efficacy. Recommendations on effectiveness precluded by large gap in available evidence. |
| **Back pain** | Usual care/  Different Psychosocial interventions. | | Moderate to severe pain  Chronic / recurrent episodes.  Non age specific/ dependent | Quality of life  Function  Pain | Long & Short term | - Combined physical and psychosocial therapies are effective with increasing level of prognostic complexities for reducing pain, and psychological distress and improving function, and return to work in people with chronic back pain compared with usual care (Henschke et al. 2011; NICE 2009; Sveinsdotirr et al. 2012). - Medium to large summary effect sizes were reported (MD -5.18; 95% CI -9.79 to -0.57, Henschke et al. 2011) on a scale of 1 to10 - Inconclusive evidence on effectiveness of psychosocial interventions especially when compared with each other ( Schaafsma et al 2003; 2013; Veehof et al. 2010). | Effectiveness of mind based stress reduction for improving pain or disability chronic LBP patientsis unclear. (Cramer et al.2012; Veehof et al. 2010).  Physiotherapy –provided operant conditioning found equally effective as other psychosocial interventions (Bunzli et al. 2011; Henschke et al. 2011). | *****Moderate evidence**  **Medium effects** | Chronic, recurrent and severe low back pain appears to benefit from psychosocial interventions in addition to usual care. However, Low specificity on components of psychosocial interventions limits recommendations on overall effectiveness. |

*Very weak evidence: Expert opinions or consensus in guidelines only / Absence of evidence in a single systematic review.

** Limited evidence: little empirical evidence from systematic reviews/evidence-based guidelines AND when there were small, inconsistent, or non-significant treatment effect sizes.

*** Moderate evidence: little empirical evidence from systematic reviews/evidence-based guidelines (as in limited evidence) but showing a medium to large treatment effect OR in the presence of strong empirical evidence from high quality systematic reviews, but with small or inconsistent treatment effect sizes across systematic reviews.

**** Strong evidence: strong empirical evidence from high quality systematic reviews and evidence based clinical guidelines AND medium or large effect sizes.
