# Supplementary material for: Effective treatment options for musculoskeletal pain in primary care: A systematic overview of current evidence
Source: PLoS One. 2017 Jun 22;12(6):e0178621. doi: 10.1371/journal.pone.0178621 (PMC5480856; doi:10.1371/journal.pone.0178621)
Supplement: S7 Table — (DOCX) [file pone.0178621.s009.docx]

|  | | **Compendium of evidence on analysis of effectiveness of surgery across regional musculoskeletal pain presentations** | | | | | | | |
| --- | --- | --- | --- | --- | --- | --- | --- | --- | --- |
| **Regional pain**  *(Sub-diagnosis)* | **Comparison (s)** | | **Specific patient profiles/ mediating risk factors**  *(e.g., pain severity @baseline; pain duration; previous pain episodes; age; movement restriction; baseline disability)* | **Outcomes**  *Pain*  *Functional Disability*  *& other 2 ^0^ Outcomes* | **Long term / short term** | **Results /Effect size** | **Specific Diagnostic considerations** | **Grade of evidence** | **Comments / summary of evidence** |
| **Neck Pain**   - *Whiplash injury/ Whiplash associated disorders (WAD)* - *Non-specific neck pain* - *Acute torticollis* - *Cervical radiculo-pathy.* | Conservative treatments | | N/A | Pain  Functional Disability  Quality of life | Long term | Surgical interventions (e.g. radiofrequency neurotomy, cervical discectomy and fusion) have weak or limited efficacy for improving recovery from neck pain diagnosis compared to conservative options (Teasell et al. 2010). | For cervical radiculopathy, the evidence for the effectiveness of surgery compared to conservative management is limited to minority of people (as low as 8%) with nerve root pain, persistent or debilitating pain combined with loss of power or sensation (MOM 2014; Nikolaidis et al. 2010). | ****Limited evidence**  **Small effects** | Surgical interventions compared to conservative options have limited indications for most neck pain management.  When implicated, choices of technique and or procedures are based on clinical judgement (MOM, 2014) as current evidence on superiority of procedures and techniques is limited. |
| **Shoulder pain**   - *General shoulder pain* - *Rotator cuff disorders* - *Shoulder impingement syndrome* - *Frozen shoulder/Adhesive Capsulitis* - *Acromioclavi-cular joint disorder* | Conservative treatments  Alternative surgical procedures and techniques | | Evidence across a wide range of patient prognostic profiles including acute and chronic situations, recurrent shoulder pain diagnosis, high to low baseline pain and disability | Pain  Function | Long & short term | Surgery does provide short-term benefit for pain, ROM and function (weak to moderate effect sizes and usually in combination with rehabilitation programmes).  In the long term, surgical procedures appears to confer no added benefits on functional and clinical outcomes compared with conservative treatments (Coghlan et al 2008, Buchbinder et al 2008, Dorrestijn et al 2009, Gebremariam et al 2011; Grant et al. 2013; Huisstede et al 2011, Maffulli 2012; Singh 2010; Tamaoki 2010). | On the basis of expert opinions, early surgical repair of full rotator cuff tears, acute dislocations of the AC joint., especially among the young and physiologically active are recommended (MoM 2014; NZGG, 2004) | ****Limited evidence**  **Small effects** | Considering overall effectiveness, surgical management is not superior to conservative treatment  When strongly indicated, evidence does not suggest any significant differences in favour of any particular surgical technique or procedure. |
| **Back pain** | Usual care/ Conservative treatments | | N/A | Pain  Functional Disability  Quality of life | Long & short term | - Surgical procedures for lumbar disc prolapse and degenerative lumbar spondylosis lead to marginal improvement in clinical outcomes compared to conservative procedures (Gibson et al. 2005; 2007; Jacobs et al 2012). | N/A | ****Limited evidence**  **Small effects** | - Insufficient evidence to claim sustained long-term improvement over the lifetime and may only be cost-effective for highly selected patients. |
| **Knee Pain**   - *Overuse injuries / tendonitis* - *Patellofemoral syndrome* - *Meniscal tears; Ligament stress / strain & Soft tissue injuries* - *Knee Bursitis* - *Degenerative knee pain / Osteoarthritis* | Usual care/ Conservative treatments | | Examined across a wide range of patient prognostic profiles including acute and chronic situations, high to low baseline pain and disability | Pain  Functional Disability | Long & short term | - Surgical Rx is effective for reducing pain and improving function, but it is only beneficial in the presence of 2nd degree pathology, substantial pain and disability or when symptoms are refractory to conservative treatment options (Brouwer et al 2005; MoM 2014). | - Arthroscopic debrigement & joint lavage has no benefit (laupattarakasem et al, 2014; Reinchenbach et al, 2010). - Evidence for the cost- effectiveness and clinical outcomes of surgery is substantiated in the presence of extensive meniscal tears (NZGG 2004). - Meniscal repairs have better long-term outcomes (but higher risk of reoperation rate) than meniscectomies (Paxton et al. 2011). - Surgical excision may be beneficial, especially in recurrent cases of acute bursitis (MoM 2014). | ***** Moderate evidence**  **Small to medium effects** | - There is currently no evidence of the superior efficacy of surgery over conservative treatment and there is so far no justification for conclusion on the comparative effectiveness of specific surgical techniques. |

*Very weak evidence: Expert opinions or consensus in guidelines only / Absence of evidence in a single systematic review.

** Limited evidence: little empirical evidence from systematic reviews/evidence-based guidelines AND when there were small, inconsistent, or non-significant treatment effect sizes.

*** Moderate evidence: little empirical evidence from systematic reviews/evidence-based guidelines (as in limited evidence) but showing a medium to large treatment effect OR in the presence of strong empirical evidence from high quality systematic reviews, but with small or inconsistent treatment effect sizes across systematic reviews.

**** Strong evidence: strong empirical evidence from high quality systematic reviews and evidence based clinical guidelines AND medium or large effect sizes.
